# Systematic review of the epidemiological evidence comparing lung cancer risk in smokers of mentholated and unmentholated cigarettes

**DOI:** 10.1186/1471-2466-11-18

**Published:** 2011-04-18

**Authors:** Peter N Lee

**Affiliations:** 1 P.N. Lee Statistics and Computing Ltd, Surrey, UK

## Abstract

**Background:**

US mentholated cigarette sales have increased considerably over 50 years. Preference for mentholated cigarettes is markedly higher in Black people. While menthol itself is not genotoxic or carcinogenic, its acute respiratory effects might affect inhalation of cigarette smoke. This possibility seems consistent with the higher lung cancer risk in Black men, despite Black people smoking less and starting smoking later than White people. Despite experimental data suggesting similar carcinogenicity of mentholated and non-mentholated cigarettes, the lack of convincing evidence that mentholation increases puffing, inhalation or smoke uptake, and the similarity of lung cancer rates in Black and White females, a review of cigarette mentholation and lung cancer is timely given current regulatory interest in the topic.

**Methods:**

Epidemiological studies comparing lung cancer risk in mentholated and non-mentholated cigarette smokers were identified from MedLine and other sources. Study details were extracted and strengths and weaknesses assessed. Relative risk estimates were extracted, or derived, for ever mentholated use and for long-term use, overall and by gender, race, and current/ever smoking, and meta-analyses conducted.

**Results:**

Eight generally good quality studies were identified, with valid cases and controls, and appropriate adjustment for age, gender, race and smoking. The studies afforded good power to detect possible effects. However, only one study presented results by histological type, none adjusted for occupation or diet, and some provided no results by length of mentholated cigarette use.

The data do not suggest any effect of mentholation on lung cancer risk. Adjusted relative risk estimates for ever use vary from 0.81 to 1.12, giving a combined estimate of 0.93 (95% confidence interval 0.84-1.02, n = 8), with no increase in males (1.01, 0.84-1.22, n = 5), females (0.80, 0.67-0.95, n = 5), White people (0.87, 0.75-1.03, n = 4) or Black people (0.90, 0.73-1.10, n = 4). Estimates for current and ever smokers are similar. The combined estimate for long-term use (0.95, 0.80-1.13, n = 4) again suggests no effect of mentholation.

**Conclusion:**

Higher lung cancer rates in Black males cannot be due to their greater preference for mentholated cigarettes. While some study weaknesses exist, the epidemiological evidence is consistent with mentholation having no effect on the lung carcinogenicity of cigarettes.

## Background

According to Giovino *et al. *[1], who summarized evidence from over 50 countries, the market share of mentholated cigarettes is relatively high in the Philippines (60%), Cameroon (35-40%), Hong Kong (26%), the United States (26%) and Singapore (22%), though it is less than 5% in half the countries considered. They reported that, in the United States, Newport brands accounted for 29.7% of all menthol sales, with Kool (10.8%), Marlboro menthol (10.5%) and Salem (10.3%) about equally distributed. According to Hebert and Kabat [2], mentholated cigarettes were introduced in the 1930s, but did not exceed 3% of the total US market until 1949, when a slow but steady rise in market share began. The market share was 16% in 1963, rose to a peak of 29% in 1979, and then declined somewhat, to 25%, in 1994-1998. The same authors [3] noted that about one third of the total production of menthol worldwide is used in the tobacco industry.

There has been increasing regulatory interest in the possible direct or indirect contribution of cigarette additives generally to the occurrence of those diseases resulting from cigarette smoking. This interest not only concerns whether added ingredients may alter the inherent disease risks of smoking, but also includes consideration of whether ingredients may render cigarettes more palatable, so as to increase initiation of smoking or decrease the ability of smokers to quit. The Family Smoking Prevention and Tobacco Control Act, signed into law in the U.S.A. in mid-2009, included a specific provision to ban characterizing flavouring ingredients other than menthol from use in cigarettes. A further review of the exemption of menthol is ongoing at the time of writing, with an advisory report and opinion to be provided to the U.S. Food and Drug Administration by the Tobacco Products Scientific Advisory Committee (TPSAC) in 2011 (http://www.fda.gov/AdvisoryCommittees/CommitteesMeetingMaterials/TobaccoProductsScientificAdvisoryCommittee/default.htm). Similar questions on whether added ingredients may increase the risks, attractiveness or addictiveness of tobacco products have also been posed by the European Commission to the EU Scientific Committee on Emerging and Newly-Identified Health Risks (SCENIHR) (http://ec.europa.eu/health/scientific_committees/consultations/public_consultations/scenihr_cons_12_en.htm), and the process of public consultation on the SCENIHR pre-consultation opinion on added ingredients is presently ongoing.

The principal objective of this paper is to carry out a systematic review, conducted according to the PRISMA guidelines [4] of the epidemiological evidence comparing the risk of lung cancer in smokers of mentholated and non-mentholated cigarettes. As background to this assessment it is helpful to start by summarizing other relevant evidence related to menthol itself and to mentholated cigarettes.

### Menthol

Menthol (C_10_H_19_OH) is a monocyclic terpene alcohol occurring as four pairs of optical isomers. (-) - Menthol, often referred to as l-menthol, is the isomer most widely seen in nature, and is the one assumed by the name menthol. It occurs naturally in over 100 essential oils [5], in high concentrations in peppermint oil (from *Mentha piperita*) and cornmint, or Japanese, mint oil (from *Mentha arvensis*), being readily extracted from the plant by steam distillation [6]. It has a characteristic peppermint odour and gives a cooling sensation when applied to skin and mucosal surfaces [6]. It has been widely used for many years, and there seems no reason for concern that it is carcinogenic or genotoxic [7,8]. A recent review [9] notes that menthol is currently approved by the US FDA for use in over-the-counter lozenges, topical preparations and vapour inhalation products based on its antipuritic and antitussive properties, and is widely used as a food flavouring, for which it is declared "Generally Recognized as Safe" (GRAS).

Eccles [6] has reviewed the evidence relating to possible effects of menthol, including nasal decongestant activity, inhibition of respiratory reflexes, antitussive efficacy, effects on mucus production and mucociliary clearance, improvement of pulmonary function and enhancement of sensations in the oral cavity. Although the evidence is not always very clear, it suggests that menthol has an acute effect on the mouth, nose and respiratory tract and certainly leaves open the possibility that menthol in cigarettes might affect puff volume, depth of inhalation and other aspects of how a cigarette is smoked. More recently, Garten and Falkner [10] speculated that menthol might induce unconscious breath holding, so allowing for greater transfer of inhaled tobacco smoke constituents into the pulmonary blood, and hence leading to an increased dependence on nicotine and risk of tobacco attributable disease.

### Mentholated cigarettes

#### Carcinogenicity, genotoxicity and pharmacological effects

While combustion of menthol under anaerobic pyrolysis conditions can produce compounds such as 3,4-benzpyrene which are known carcinogens [11-13], menthol pyrolysis in cigarettes does not give rise to any measurable amounts of 3,4-benzpyrene [9,14-19], and there are no notable differences in benzpyrene between major US mentholated and non-mentholated cigarette brands [20].

Two studies compared mouse skin activity of condensates prepared from mentholated and non-mentholated cigarettes, but found no significant difference in either tumourigenic activity [21] or tumour promoting potential [22]. Gaworski *et al. *[5,23] reported results from two 13-week inhalation studies where rats were exposed to the smoke of cigarettes with or without added menthol, or to filtered air. In the second experiment [23], the test cigarette differed from the reference cigarette in having various other flavour ingredients added as well as menthol. In both studies, dose-related histopathological changes, mainly of respiratory tract epithelia, were found in rats exposed to mainstream smoke. However, the changes were similar in cigarettes with and without menthol, not neoplastic, and diminished significantly after a 6-week recovery period. A later 90-day study [24] also found no meaningful differences in smoke-related changes between the mentholated and non-mentholated cigarettes. Although these results provide no concern regarding possible carcinogenic effects resulting from cigarette mentholation, it should be noted that results of long term (2 yr+) inhalation studies have never been reported.

Heck [9] recently reviewed evidence comparing the *in vitro *cytotoxicity and genetic toxicity of smoke condensates and gas-phase material from mentholated and non-mentholated cigarettes, finding no meaningful differences.

#### Effects on smoking characteristics

Many studies [25-44] have compared mentholated and non-mentholated cigarette smokers on one or more of the most commonly studied smoking indices: number of puffs, puff volume, carbon monoxide (CO) and cotinine. Nearly all the studies cited took into account possible racial differences. Of seven studies investigating number of puffs, four [25-28] found no significant effect of mentholation, and three [29-31] reporting significantly fewer puffs when smoking mentholated cigarettes. Of six studies on puff volume, two studies [26,27] found no effect, three [29-31] a significant decrease when smoking mentholated cigarettes, and one [32] a significant increase. While three studies [26,33,34] found an increase in CO level associated with mentholation (and one [30] claimed an increase not apparent from the analyses presented), many studies [25,28,29,31,35-39] found no effect of mentholation, and one [27] reported reduced CO in mentholated cigarette smokers. Three studies [32-34] reported a significant increase in cotinine level associated with mentholation, but nine studies [27,37-44] did not, one of these[39] being based on more subjects (3,341) than all the other studies combined. Most of this evidence was considered in a review in 2007 [45] which, taking also into account other evidence (e.g. on heart rate, blood pressure, total particulate matter, and serum thiocyanate) concluded that "Taken as a whole, the data provide little consistent support for the idea that mentholation may affect how a cigarette is smoked so as to increase uptake of toxic smoke constituents". A more recent review reached similar conclusions [9].

Five studies have compared age of starting to smoke in mentholated and non-mentholated cigarette smokers. No significant differences were seen in the largest study [46], which involved over 10,000 subjects and adjusted for race, in two studies in Black people [47,48], or in two other studies [49,50] which did not adjust for race.

Two very large studies [51,52], each of over 10,000 subjects, reported that, in both Black and White people, mentholated cigarette smokers smoked significantly fewer cigarettes per day than did non-mentholated cigarette smokers. Reduced cigarette consumption has also been reported in four other studies [39,50,53,54], though two [53,54] did not adjust for race and one [39] reported that the reduction was only evident in Black people, with an increase in White people. However, no significant difference was seen in another study of over 10,000 subjects [46] which did adjust for race, or in other studies, three conducted in Black people [47,48,55], one mainly in White people [56], and two [49,57] which did not take race into account.

Sixteen studies have reported results on quitting, race being accounted for in all but one [53]. The two largest [46,52] found no difference by mentholation. No significant differences were also seen in eight other studies [47,49,54,56,58-61]. However, reduced quitting was reported in six studies [48,50,53,62-64], though in two of these [62,63], both trials of smoking cessation, the lower quit rate in mentholated cigarette smokers was only seen in the first month or so, longer follow-up of the subjects finding no difference in quit rate. In one study [64], the reduced rate of quitting was only evident in Black and Hispanic people combined, a significantly increased rate of quitting being seen in White people.

The evidence summarized above does not suggest that mentholation of cigarettes is associated with an earlier (or later) age of starting smoking or any increase in cigarette consumption per smoker. Any effect on quitting, if it exists, seems probably quite small.

### Differences between Black and White people in the US

#### Relative use of mentholated cigarettes

Among United States smokers, preference for mentholated cigarettes is much greater in Black than White people. Data from the 2008 National Survey on Drug Use and Health (NSDUH) presented in Table 1 show the difference is clearly seen in both sexes and in all age groups (except for 12-17 year-old females). It can also be seen in earlier NSDUH surveys (see [45]). Despite this marked difference, the much higher proportion of White people implies that many more White than Black people smoke mentholated cigarettes. The tendency for Black people to prefer mentholated cigarettes has long been evident. For example, in a national study in 1986 cited by the US Surgeon-General [65], 75.5% of Black and 23.1% of White smokers used mentholated cigarettes.

**Table 1:** Relative use of mentholated cigarettes in US Black and White people in 2008 by gender and age^a^

		**% of current smokers using mentholated cigarettes**^**b**^	
Gender	Age	White people	Black people
Male	12-17	45.1	79.1
	18-25	29.5	86.5
	26-34	22.7	89.6
	35-49	14.9	87.9
	50+	20.2	70.4
	All ages	21.8	83.5
			
Female	12-17	46.9	51.8
	18-25	36.0	88.7
	26-34	24.1	94.0
	35-49	28.0	95.1
	50+	28.6	89.4
	All ages	29.6	90.9

^a ^Derived from the NSDUH (http://www.icpsr.umich.edu/cgi-bin/SDA/SAMHDA).

^b ^Current smokers were asked whether they had smoked mentholated or regular cigarettes most in the past 30 days.

#### Relative lung cancer rates in Black and White people in the US

United States data for 2001 to 2005 combined [66] show that age-adjusted incidence rates were 36% higher in Black men than in White men, and mortality rates 31% higher. In females, however, incidence rates were 0.5% lower and mortality rates 5% lower. Table 2 shows US lung cancer mortality rates by race, gender, year (2000, 2005), and age. In both sexes, the ratio of rates in Black people compared to White people tends to decline with age, with the clearest excesses seen in younger males (especially in 2000), lesser excesses seen in older males and younger females, and no excess seen in older females. Although it is often suggested (e.g. [17,67-69]) that the greater preference of Black people for mentholated cigarettes might help explain their higher lung cancer rates, the greater preference is similarly evident in both genders, but the excess lung cancer rate is only in males.

**Table 2:** Relative lung cancer mortality rates in US White and Black people by gender, year and age^a^

			White people		Black people		Black/White
Gender	Year	Age	Deaths	Rate	Deaths	Rate	Rate
Male	2000	35-44	1133	6.1	361	13.3	2.19
		45-54	5588	35.7	1383	70.7	1.98
		55-64	15173	150.7	2436	223.4	1.48
		65-74	27516	374.7	3340	488.7	1.30
		75-84	23406	529.7	2118	641.3	1.21
							
	2005	35-44	943	5.3	203	7.6	1.43
		45-54	5803	33.2	1334	56.9	1.71
		55-64	15510	123.4	2721	199.1	1.61
		65-74	24627	331.7	3011	408.6	1.23
		75-84	24598	519.7	2066	564.3	1.09
							
Female	2000	35-44	957	5.2	222	7.3	1.40
		45-54	3941	24.8	746	32.8	1.33
		55-64	10304	96.0	1289	95.3	0.99
		65-74	18663	213.1	1885	194.1	0.91
		75-84	18299	272.5	1315	224.2	0.82
							
	2005	35-44	907	5.2	184	6.1	1.17
		45-54	4230	24.0	914	33.5	1.40
		55-64	10947	82.6	1489	87.8	1.06
		65-74	17883	207.1	1899	184.5	0.89
		75-84	19816	288.3	1632	253.2	0.88

^a^Derived from the National Center for Health Statistics website (http://wonder.cdc.gov).

#### Differences in smoking habits between Black and White people in the US

In the United States, there are other relevant differences in smoking habits between White and Black smokers. Table 3 summarizes findings from the National Health Interview Survey for 2006. These are consistent with other evidence (e.g. [17,46,65,70-72]) that Black people have a slightly higher prevalence of smoking, a later age of starting to smoke, a lower daily cigarette consumption per smoker, a lower propensity to quit, and a preference for cigarettes higher in tar and nicotine. According to Novotny *et al. *[70] the difference in smoking prevalence by race disappears if adjustment is made for occupation, education and other socioeconomic and demographic factors, but the differences in amount smoked per smoker and probability of quitting do not.

**Table 3:** Differences in cigarette smoking habits between White and Black adults - findings from the National Health Interview Survey for 2006^a^

	Males		Females	
	White people	Black people	White people	Black people
**Among the whole population**				
Ever smoked (100 cigarettes)	49.9%	42.8%	38.2%	28.7%
Current smoker^b^	23.7%	27.0%	18.5%	18.9%
**Among ever smokers**				
Current smoker^b^	47.5%	63.0%	48.4%	66.0%
Age of starting to smoke (mean, yrs)	16.9	18.4	17.9	19.2
**Among current smokers^b^**				
Number smoked per day (mean)	16.1	11.4	13.9	10.0

^a^Derived from http://www.icpsr.umich.edu/cgi-bin/SDA/ICPSR.

^b^Includes current every day and some day smokers.

The evidence is also consistent that Black smokers have higher cotinine levels than White smokers [44,73-77]. In one large study [73] serum cotinine levels averaged 210.2 ng/ml in White men, 244.8 ng/ml in Black men, 176.4 ng/ml in White females and 251.2 in Black females. After adjusting for age, education, gender, cigarettes/day, nicotine content, years of smoking, inhalation frequency and ETS exposure, the difference was estimated to be 83.3 ng/ml, with the higher level in Black people significant for both mentholated cigarette smokers (89.0 ng/ml) and non-mentholated cigarette smokers (51.5 ng/ml).

Of the characteristics considered above, some would predict lower lung cancer rates in Black people (fewer ever smokers, later age of starting, and lesser amount smoked per smoker) and some higher rates (more current smokers, reduced quitting, higher tar level, and higher cotinine levels).

## Methods

### Selection of studies

Studies selected satisfied four conditions: based on research on humans, of cohort or case-control design, any form of lung cancer as the outcome, and risk estimates comparing mentholated and non-mentholated cigarette smokers available or able to be calculated.

Relevant publications were initially sought from a MedLine search conducted in August 2010, on "Lung cancer and (menthol or mentholated cigarettes)" limited to "Humans", from recent reviews relating to mentholated cigarettes [9,45], and from reference lists of relevant publications identified. Subsequently, in February 2011, the MedLine search was repeated, and additional papers sought from further similar searches conducted using Google Scholar, Scopus, Scirus, Science Direct and Academic Search Complete, and also from reference lists of new relevant publications identified.

### Data extraction

From each publication details extracted included:

• study design (prospective cohort, hospital case-control, population case-control)

• study location and timing

• sexes, races and age groups studied

• whether current or ever smokers were studied

• other inclusion criteria

• cases - definition, number studied

• controls (for case-control studies) - definition, matching to cases, number studied

• at risk population (for cohort studies) - definition, number studied

• questions asked relating to mentholation of cigarettes (and brands of cigarettes smoked) and the variables used to quantify exposure

• statistical methods used for analysis

• adjustment variables considered

• availability of results by histological type, and

• main conclusions reached by the authors.

### Relative risk estimates

For simplicity, the term "relative risk" (RR) is used generically in this paper to include various estimators of it, including the odds ratio and the hazard ratio.

Studies varied in the extent to which they reported estimates of RR with a 95% confidence interval (CI). The reported estimates were supplemented by derived estimates, in an effort to present, as far as possible, estimates by extent of mentholated cigarette use and overall, by gender separately and combined, by race separately and combined, by age groups separately and combined, by histological type of lung cancer separately and combined, in current or ever smokers separately and combined, and also by extent of adjustment for confounding variables. The full set of RR (CI) estimates for each study, reported and derived, are given in the tables which describe the results for each individual study. Details of how the various estimates were derived are given in additional file 1: Methods for deriving RR estimates. Three main techniques were used: Unadjusted estimates were derived from numbers of cases and controls using standard methods [78], independent estimates were combined using fixed-effect meta-analysis [79], and non-independent estimates (relative to a common comparison group) were combined using the method of Hamling *et al*. [80].

### Summary of study characteristics, strengths and weaknesses

The features of the studies were first described study-by-study and then summarized in a table. For each of a number of features (study type, location, timing, possible overlaps between the studies, number of cases, adequacy of cases, adequacy of controls, reliability of data collected, adjustment for potential confounding variables, and statistical methods used), the propensity for bias was considered, and strengths and weaknesses of the overall evidence of specific studies evaluated. Study quality was also independently assessed, using the nine-point Newcastle-Ottawa Quality Assessment Scale (NOS) [81], by the author and a colleague (K.J. Coombs), with any discrepancies resolved by discussion.

### Meta-analyses

Fixed-effect and random-effects meta-analyses were conducted, and heterogeneity chisquared statistics estimated, using standard methods [79]. The principal comparison was between smokers who had ever or never used mentholated cigarettes, though as this was not available in some studies RRs for similar comparisons (e.g. usual brand mentholated cigarettes or not; current brand mentholated or not) were also included in the meta-analysis. A secondary comparison used the same comparison group (never or non-current mentholated cigarettes), but assessed risk relating to long-term use of mentholated cigarettes, using the maximum extent of exposure available for the study (e.g. 20+ years use or 32+ pack-years use). The main analyses used combined estimates for the whole population studied, but subgroup analyses give results by gender, race, smoking status, study quality, study size (number of cases in mentholated cigarette smokers), study design, and publication date. Sensitivity analyses excluded the smaller of these studies, where there was a possible overlap in cases between studies. For the main analysis, publication bias was investigated by Egger's test [82] and by a funnel plot, in which the logarithm of the RR is plotted against its (inverse-variance) weight.

### Checking

Dr. J.S. Fry independently checked the extraction of data from the source publications, the derivation of additional relative risk estimates and the conduct of the meta-analyses. Any differences found were resolved in discussion with the author.

## Results

### Studies identified

**Figure 1:**
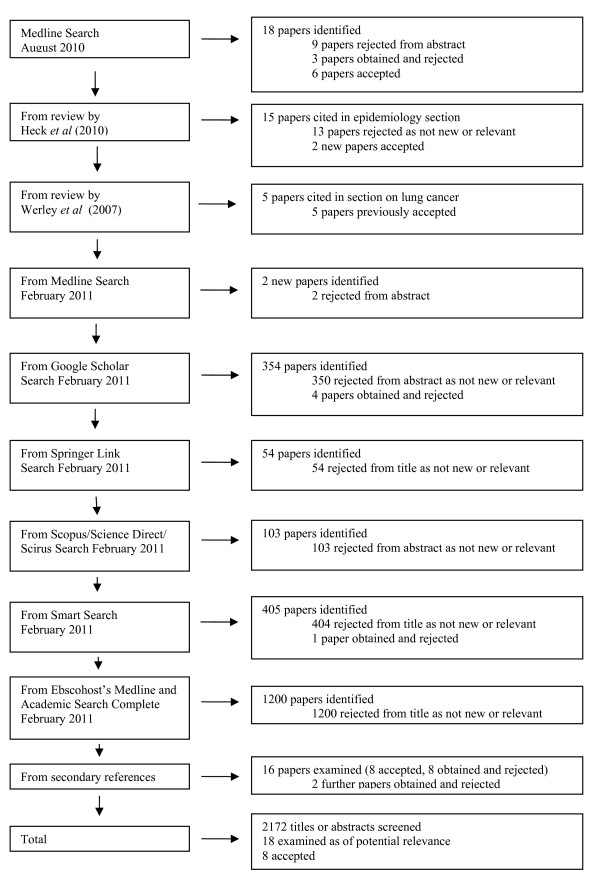
**Flow diagram for literature search**. The diagram shows the number of papers identified at various stages of the search, and the numbers rejected and accepted. (Note that in the last box, showing the total number of titles as abstracts screened, the figure of 2172 double-counts papers identified in more than one search.)

Eight epidemiological studies were identified that provided evidence on the relative risk of lung cancer associated with cigarette mentholation. Six [51,53,56,68,69,83] were identified from the MedLine search conducted in August 2010, with a further two [84,85] referred to in the review by Heck [9]. No further relevant studies were identified from other sources, including the later searches conducted in February 2011. Fuller details of the sequence of searches made, and the number of papers identified, rejected and accepted at the various stages are shown in Figure 1.

Apart from a case-control study in Germany, reported only as an abstract [84], all the studies were conducted in the USA. Two of these are of prospective cohort design [51,56], the remaining five being case-control studies [53,68,69,83,85]. In the following sections, the eight studies are described in chronological order of publication and their relevant results summarized.

### Study descriptions and results

#### American Health Foundation multicentre case-control study

The first paper [68] to report results relating use of mentholated cigarettes to risk of lung cancer, published in 1991, was based on data from a long-term multicentre hospital case-control study. This involved patients with tobacco-related cancers and controls - hospitalized patients with conditions thought not associated with smoking - matched to the cases on age, gender, race, hospital and date of interview. The analyses were restricted to current smokers of cigarettes (defined as subjects who had smoked in the year preceding diagnosis) who were interviewed between 1985 and 1990 in one of eight hospitals in four US cities. They included 588 male and 456 female histologically confirmed lung cancer cases and 914 male and 410 female controls.

All patients were interviewed in hospital using a standard questionnaire. This contained questions on the type of tobacco products used throughout life, brands of cigarettes smoked, cigarettes per day, use of filter and non-filter cigarettes, use of mentholated cigarettes, years of smoking each brand and age at initiation. Information on mentholation was obtained for each brand of cigarette reported.

Table 4 summarizes the results of unconditional logistic regression analyses used to estimate the risk of lung cancer associated with smoking mentholated cigarettes for 1-14 years or 15+ years relative to never having smoked mentholated cigarettes, with adjustment for age, race, education, cigarettes per day (of the current brand), inhalation, duration of smoking, body mass index and, where appropriate, also gender. Without adjustment for potential confounding variables, use of mentholated cigarettes was associated with a reduced risk of lung cancer, not significantly in males (RR 0.92; CI 0.73-1.17) but significantly in females (RR 0.56; CI 0.42-0.75). With adjustment for the variables noted above, however, no significant association was seen (males 1.06; 0.82-1.37, females 0.78; 0.57-1.08). Nor was any association seen when results (only available for the genders combined, and as adjusted relative risks) were considered by histological type.

**Table 4:** Risk of lung cancer by mentholated cigarette use among current smokers - American Health Foundation multicentre case control study [68]

Histological type of lung cancer				Years of use of mentholated cigarettes			
				
		No. of		**Never**^**b**^	1-14	15+	**Ever**^**c**^
				
	Gender	Cases	**Adjusted**^**a**^	RR	RR (CI)	RR (CI)	RR (CI)
Any	Male	588	No^d^	1.00	0.98 (0.72-1.33)	0.87 (0.64-1.20)	0.92 (0.73-1.17)
			Yes	1.00	1.14 (0.82-1.59)	0.98 (0.70-1.38)	1.06 (0.82-1.37)
	Female	456	No^d^	1.00	0.56 (0.38-0.83)	0.55 (0.38-0.80)	0.56 (0.42-0.75)
			Yes	1.00	0.82 (0.52-1.28)	0.76 (0.53-1.16)	0.78 (0.57-1.08)
	Combined	1044	No^d^	1.00	0.79 (0.62-1.01)	0.72 (0.57-0.92)	0.76 (0.63-0.91)
			Yes^d^	1.00	1.02 (0.78-1.33)	0.88 (0.68-1.14)	0.94 (0.77-1.15)
Squamous cell carcinoma	Combined	268	Yes	1.00	1.17 (0.78-1.78)	0.92 (0.60-1.42)	1.04 (0.75-1.44)
Small cell carcinoma	Combined	131	Yes	1.00	0.80 (0.43-1.48)	0.86 (0.49-1.51)	0.83 (0.53-1.30)
Large cell carcinoma	Combined	106	Yes	1.00	1.99 (0.73-5.41)	0.84 (0.27-2.61)	1.28 (0.56-2.91)
Adenocarcinoma	Combined	400	Yes	1.00	0.98 (0.68-1.42)	0.95 (0.66-1.36)	0.96 (0.73-1.28)

^a ^Yes = adjusted for age, race, education, cigarettes per day, inhalation, duration of smoking and body mass index. Combined gender analyses are also adjusted for gender.

^b ^Or less than 1 year mentholated use.

^c ^At least 1 year mentholated use.

^d ^RRs and CIs estimated as described in additional file 1: Methods for deriving RR estimates.

The authors concluded that: "Use of mentholated cigarettes was not associated with increased risk of lung cancer or of specific histological types of lung cancer in this study" and noted that "If our results are confirmed by other researchers, the implication would be that use of mentholated cigarettes does not explain Black-White differences in lung cancer incidence or time trends."

#### Kaiser Permanente prospective study

The second relevant paper identified [51], published in 1995, was based on data from the Northern California Kaiser Permanente Medical Care Program in Oakland, California. The study involved 5771 males and 5990 females aged 30 to 89 years who underwent a multiphasic health check-up between 1979 and 1985, reported they were then current cigarette smokers who had smoked for at least 20 years, and provided details of the mentholation status of the brand of cigarettes they usually smoked. Follow-up for lung cancer was determined up to the end of 1991 with 318 incident cases identified, 93 in mentholated cigarette users, 225 in non-mentholated cigarette users, 168 in males and 150 in females.

As shown in Table 5, risks for males were somewhat higher in users than in non-users in every age group and after adjustment for age, the RR could be estimated as 1.40 (CI 1.01-1.94). This marginally statistically significant difference was also seen when adjustment was made for age, race and education as well as years of smoking and cigs/day (using a Cox proportional hazards model), the RR given being 1.45 (1.03-2.02). In males, duration of mentholated cigarette use also showed a significant (p = 0.02) trend with risk of lung cancer. In females, however, risk was somewhat less in users than in non-users with the RR 0.71 (0.50-1.00) after adjustment for age and 0.75 (0.51-1.11) after additional adjustment for race, education, years of smoking and cigs/day. When data for males and females were combined, with additional adjustment for gender, no significant association was seen.

**Table 5:** Risk of lung cancer by mentholated cigarette use among current smokers for 20 years or more - Kaiser Permanente prospective study [51]

				Duration of mentholated cigarette use (years)				
				
				0	1-9	10-19	20+	Any
				
Gender	Age	**No. of cases**^**a**^	Adjusted	RR	RR (CI)	RR (CI)	RR (CI)	RR (CI)
Male	< 50	6 (3)	No	1.0	-	-	-	2.2 (0.5-11.1)
	50-64	82 (24)	No	1.0	-	-	-	1.1 (0.7-1.8)
	65-74	60 (22)	No	1.0	-	-	-	1.7 (1.0-2.9)
	75+	20 (6)	No	1.0	-	-	-	1.7 (0.6-4.3)
	All	168 (55)	Age^b^	1.00	-	-	-	1.40 (1.01-1.94)
	All	160 (51)^c^	Age, others^d^	1.00	-	-	-	1.45 (1.03-2.02)
	All	158 (57)^e^	Age, others^d^	1.00	1.10 (0.65-1.87)	1.32 (0.84-2.08)	1.59 (0.96-2.63)	-
								
Female	< 50	11 (2)	No	1.0	-	-	-	0.3 (0.1-1.6)
	50-64	61 (19)	No	1.0	-	-	-	0.8 (0.5-1.4)
	65-74	50 (10)	No	1.0	-	-	-	0.6 (0.3-1.1)
	75+	28 (7)	No	1.0	-	-	-	0.9 (0.4-2.1)
	All	150 (38)	Age^b^	1.00	-	-	-	0.71 (0.50-1.00)
	All	138 (34)^c^	Age, others^d^	1.00	-	-	-	0.75 (0.51-1.11)
	All	132 (42)^c^	Age, others^d^	1.00	0.72 (0.38-1.39)	1.01 (0.61-1.69)	0.70 (0.40-1.23)	-
								
Combined	< 50	17 (5)	Gender^b^	1.00	-	-	-	0.73 (0.26-2.04)
	50-64	143 (43)	Gender^b^	1.00	-	-	-	0.95 (0.67-1.35)
	65-74	110 (32)	Gender^b^	1.00	-	-	-	1.12 (0.74-1.69)
	75+	48 (13)	Gender^b^	1.00	-	-	-	1.17 (0.62-2.21)
	All	318 (93)	Gender, age^b^	1.00	-	-	-	1.02 (0.80-1.29)
	All	298 (85)^c^	Gender, age, others^b,d^	1.00	-	-	-	1.09 (0.85-1.41)
	All	290 (99)^c^	Gender, age, others^b,d^	1.00	0.93 (0.62-1.40)	1.17 (0.84-1.65)	1.10 (0.76-1.60)	-

^a ^The first number includes non-mentholated cigarette smokers and the bracketed number is for mentholated cigarette smokers only

^b ^RRs and CIs derived as described in additional file 1: Methods for deriving RR estimates.

^c ^There were fewer subjects in the analyses adjusted for age and other variables than for those adjusted for age only, presumably because of missing data for the other variables.

^d ^The other adjustment factors were race, education, years of smoking and number of cigarettes per day.

^e ^The lesser total cases in this analysis than the previous one is presumably because of missing data on duration of mentholated cigarette use. However, it is unclear why there were more cases who smoked mentholated cigarettes.

The authors noted that additional adjustment for aspects of smoking other than years of smoking and cigs/day did not substantially alter the estimate of relative risk for mentholated cigarette use, and concluded that: "This study suggests there is an increased risk of lung cancer associated with mentholated cigarette use in male smokers but not in female smokers."

#### Los Angeles County case-control study

Whereas the first two papers limited attention to current cigarette smokers, the third [69] also considered former smokers. This study, conducted in 1991-1994 in Los Angeles County in California and reported in 1999, was of population case-control design. Cases were histologically confirmed and identified within seven months of diagnosis. Controls under age 65 were randomly selected from licensed drivers, whilst those over age 65 were randomly selected from Medicare Beneficiaries. Cases and controls were frequency matched on age, gender and race. Cases and controls had to be resident in Los Angeles County, aged 40-84, able to complete a questionnaire in English, be Caucasian (non-Hispanic) or African American people, and with no previous cancer (other than non-melanoma skin cancer). Subjects were interviewed regarding known and possible risk factors for lung cancer, including smoking history, occupational exposures, ETS exposure and family history of lung cancer. Menthol smoking was classified based on response to the question "On average over your lifetime, out of every 100 cigarettes you smoked, how many were menthol?".

Of 859 cases and 3193 potentially eligible controls, 353 cases and 724 controls were available for interview and provided smoking information including menthol status. The analysis was restricted to the cases (202 males and 135 females) and controls (349 males and 129 females) who had ever smoked (as many as 100 cigarettes in their life). The analyses presented used unconditional logistic regression, with adjustment for age, race, gender, total pack-years and years since quitting smoking. Other potential confounding variables (fruits, vegetables, occupational exposures, family history and ETS) had no appreciable influence on the association with mentholated cigarette smoking and were therefore not included in the regression models.

RRs and CIs were presented by pack-years of mentholated smoking (Table 6), percentage of mentholated cigarettes smoked (Table 7) and type of cigarette smoker (Table 8). RRs and CIs are available (or can be derived) which are unadjusted, adjusted for the matching factors gender, age and race, or adjusted for the matching factors as well as for total pack-years and years since quitting smoking. No results are available for current and former smokers separately. Results by pack-years of mentholated smoking are separately available by gender, and by race.

**Table 6:** Risk of lung cancer by pack-years of mentholated cigarette use among ever smokers - Los Angeles County case-control study [69]

			Pack-years of mentholated smoking				
			
Gender/Race	No of cases		0	1-15	16-31	32+	**Any**^**a**^
			
		Adjusted	RR	RR (CI)	RR (CI)	RR (CI)	RR (CI)
Male/Both	202	No	1.00	0.63 (0.42-0.95)	1.40 (0.69-2.84)	2.60 (1.33-5.08)	0.92 (0.65-1.31)
		Age, race^b^	1.00	0.62 (0.41-0.93)	1.35 (0.67-2.73)	2.52 (1.29-4.92)	0.90 (0.64-1.28)
		Age, race and others^c^	1.00	0.87 (0.57-1.37)	1.21 (0.56-2.62)	1.48 (0.71-3.05)	1.00 (0.68-1.48)
Female/Both	135	No	1.00	0.90 (0.52-1.57)	0.83 (0.36-1.96)	0.90 (0.38-2.15)	0.89 (0.55-1.44)
		Age, race^b^	1.00	0.87 (0.50-1.52)	0.68 (0.29-1.60)	0.72 (0.30-1.71)	0.80 (0.49-1.29)
		Age, race and others^c^	1.00	1.58 (0.77-3.22)	0.51 (0.19-1.34)	0.41 (0.15-1.11)	0.88 (0.50-1.57)
Both/White people^d^	181	No	1.00	0.64 (0.41-1.00)	1.35 (0.58-3.11)	1.80 (0.87-3.75)	0.86 (0.59-1.26)
		Age, gender^b^	1.00	0.68 (0.44-1.06)	1.41 (0.61-3.25)	1.78 (0.86-3.70)	0.90 (0.61-1.31)
		Age, gender and others^c^	1.00	1.01 (0.61-1.68)	1.01 (0.41-2.47)	1.06 (0.47-2.36)	1.02 (0.66-1.58)
Both/Black people^e^	156	No	1.00	0.71 (0.43-1.16)	0.91 (0.44-1.89)	1.56 (0.72-3.37)	0.85 (0.55-1.32)
		Age, gender^b^	1.00	0.66 (0.40-1.08)	0.77 (0.37-1.60)	1.46 (0.68-3.16)	0.78 (0.51-1.21)
		Age, gender and others^c^	1.00	0.96 (0.54-1.70)	0.69 (0.30-1.60)	0.90 (0.38-2.12)	0.89 (0.53-1.47)
Both/Both	337	No	1.00	0.72 (0.52-1.00)	1.20 (0.70-2.06)	1.81 (1.07-3.07)	0.93 (0.70-1.23)
		Age, race and gender^b^	1.00	0.70 (0.51-0.97)	1.04 (0.61-1.79)	1.64 (0.97-2.76)	0.87 (0.66-1.15)
		Age, race, gender and others^c^	1.00	1.05 (0.72-1.54)	0.92 (0.50-1.68)	0.95 (0.53-1.70)^f^	1.00 (0.72-1.40)

^a^RRs and CIs for any use estimated as described in additional file 1: Methods for deriving RR estimates.

^b ^The width of the estimated CI is taken to be the same as the width of the unadjusted estimates, so may be slightly too narrow.

^c ^The other adjustment factors were total pack-years and years since quitting.

^d ^White people are defined as Caucasian.

^e ^Black people are defined as African-American.

^f ^Similarly adjusted RRs and CIs are also available for 32-53 pack-years, 0.76 (0.37-1.59) and 54+ pack-years 1.38 (0.56-3.40). For 54+ pack-years these estimates did not vary between current smokers (RR 1.23, 0.36-1.42) and former smokers (RR 1.78, 0.44-7.19).

**Table 7:** Risk of lung cancer by percentage of mentholated cigarettes smoked - Los Angeles County case-control study [69]

			Percentage of mentholated smoking								
			
Gender/Race	No of cases		0		1-19		20-74		75-100		**Any**^**a**^
			
		Adjusted	RR		RR (CI)		RR (CI)		RR (CI)		RR (CI)
Both/Both^b^	337	No	1.00		0.87 (0.59-1.29)		0.84 (0.55-1.29)		1.09 (0.73-1.63)		0.93 (0.70-1.23)
		Age, race^c ^and gender	1.00		0.94 (0.64-1.39)		0.73 (0.48-1.12)		0.94 (0.63-1.41)		0.87 (0.66-1.15)
		Age, race, gender and others^d^	1.00		1.11 (0.71-1.72)		0.90 (0.55-1.45)		1.02 (0.65-1.63)		1.01 (0.74-1.40)

^a ^RRs and CIs for any use estimated as described in additional file 1: Methods for deriving RR estimates.

^b ^Caucasian and African/American people.

^c ^The width of the estimated CI is taken to be the same as the width of the unadjusted estimates, so may be slightly too narrow.

^d ^The other adjustment factors were total pack-years and years since quitting.

**Table 8:** Risk of lung cancer by cigarette smoker type - Los Angeles County case-control study [69]

			Cigarette smoker type			
			
			Exclusive regular	Exclusive menthol	Mixed menthol/regular	**Any menthol**^**a**^
Gender/Race	No of cases	Adjusted	RR	RR (CI)	RR (CI)	RR (CI)
Both/Both^b^	337	No	1.00	1.17 (0.74-1.85)	0.86 (0.64-1.17)	0.93 (0.70-1.23)
		Age, race^c ^and gender	1.00	1.10 (0.70-1.74)	0.83 (0.61-1.13)	0.89 (0.67-1.18)
		Age, race, gender and others^d^	1.00	1.04 (0.62-1.75)	1.01 (0.71-1.42)	1.02 (0.74-1.40)

^a ^RRs and CIs for any use estimated as described in additional file 1: Methods for deriving RR estimates.

^b ^Caucasians and African/American people.

^c ^The width of the estimated CI is taken to be the same as the width of the unadjusted estimates, so may be slightly too narrow.

^d ^The other adjustment factors were total pack-years and years since quitting.

For all subjects, the RRs comparing smokers who have ever and never used mentholated cigarettes are close to 1.0, when adjustment is made for the matching factors and the smoking variables considered. Due to rounding of RRs presented by level for the three aspects of mentholated cigarette smoking, these calculated estimates are not quite the same in the three tables, being 1.00 (CI 0.72-1.40), 1.01 (0.74-1.40) and 1.02 (0.74-1.40) in Tables 6, 7 and 8 respectively. The results in Table 6 also show that the ever/never mentholated RR is quite close to 1 for males (1.00; 0.68-1.48), females (0.88; 0.50-1.57), Caucasian people (1.02; 0.66-1.58) or African-American people (0.89, 0.53-1.47). Nor is there any evidence of any variation in risk by proportion of mentholated cigarettes smoked (Table 7) or by cigarette smoker type (Table 8). After adjustment for the matching and smoking variables the RR was 1.04 (0.62-1.75) for exclusive mentholated and 1.01 (0.71-1.42) for mixed menthol/regular (non-mentholated), as compared to exclusive regular.

The only apparent suggestion of an effect of mentholated cigarette smoking is for the results by pack-years (Table 6). In interpreting these results, it is important to realise, that the RRs which are unadjusted or are adjusted only for the matching factors may be biased by the greater likelihood of inclusion of current and long term smokers in the higher pack-years categories. When adjustment is also made for total pack-years and years since quitting, this bias should mainly be removed, though there remains the possibility of some residual confounding. After this adjustment there was no evidence of any variation in risk by pack-years of mentholation for the total population or for Caucasian or African-American people separately. However, there was some evidence that risk increased with pack-years in males (RRs 1.00, 0.87, 1.21 and 1.48 for 0, 1-15, 16-31 and 32+ pack-years, trend p = 0.25) and that risk decreased with pack-years in females (RRs 1.00, 1.58, 0.51 and 0.41, trend p = 0.04).

The authors concluded that "Our results suggest that the lung-cancer risk from smoking mentholated cigarettes resembles the risk from smoking non-mentholated cigarettes. Our data do not support the hypothesis that the increased risk of lung cancer among African Americans is due to the increased prevalence of menthol smoking."

#### Slone Epidemiology Center study

The fourth paper [53], reported in 2003, also considered ever smokers. It concerned a case-control study conducted in 1981-2000 in hospitals in four eastern US states. Cases were confirmed by review of pathology reports, with the diagnoses made within 12 months of admission. Controls were admitted for conditions unrelated to smoking. Cases and controls had to be aged 40-74, have smoked cigarettes for at least 20 years and have no history of cancer. 1300 cases and 9383 controls met the initial eligibility criteria, but analysis was restricted to those cases (435 males and 208 females) and controls (2123 males and 1987 females) for whom brand information could be identified for at least 60% of the total duration of smoking (assuming that subjects with an unknown brand history before 1956 smoked non-mentholated cigarettes at that time). Analyses used unconditional logistic regression with adjustment for age, gender, race, year of interview and various smoking variables (number of years of smoking, number of cigarettes smoked per day, years since quitting smoking and proportion of years smoking filter cigarettes).

The results shown in Table 9 provide no indication of an effect of mentholation on lung cancer risk. In the most adjusted analyses, RRs show no significant effect of mentholation in males, females, White people or Black people. Overall, ever using mentholated cigarettes is associated with a RR of 0.89 (95% CI 0.89-1.14), and there is no increase for > 15 years use (0.97; 0.70-1.34). In the less adjusted analyses, RRs are always less than 1.00, and often significant, particularly in the crude analyses. This probably reflects uncontrolled confounding.

**Table 9:** Risk of lung cancer by number of years of smoking mentholated cigarettes among ever smokers for at least 20 years - Slone Epidemiology Center case-control study [53]

			Years of use of mentholated cigarettes			
			
			Never	1-15	> 15	**Ever **^**a**^
			
Gender/Race	No. of Cases	Adjusted	RR	RR (CI)	RR (CI)	RR (CI)
Male/Both	435	No^b^	1.00	0.58 (0.39-0.86)	0.62 (0.42-0.93)	0.60 (0.45-0.80)
		Age, race^c^	1.00	0.61 (0.41-0.90)	0.71 (0.48-1.06)	0.66 (0.49-0.88)
		Age, race and others^d^	1.00	0.67 (0.43-1.05)	0.91 (0.57-1.46)	0.77 (0.55-1.08)
Female/Both	208	No^b^	1.00	0.67 0.42-1.09)	0.75 (0.51-1.11)	0.72 (0.52-1.00)
		Age, race^c^	1.00	0.76 (0.47-1.23)	0.89 (0.60-1.32)	0.84 (0.60-1.16)
		Age, race and others^d^	1.00	1.14 (0.66-1.95)	1.00 (0.63-1.60)	1.05 (0.72-1.55)
Both/White people	515	No^b^	1.00	0.69 (0.49-0.98)	0.71 (0.50-1.01)	0.70 (0.54-0.91)
		Age, gender^c^	1.00	0.71 (0.50-1.01)	0.87 (0.61-1.24)	0.78 (0.60-1.01)
		Age, gender and others^d^	1.00	0.86 (0.59-1.28)	1.01 (0.68-1.51)	0.93 (0.69-1.24)
Both/Black people	128	No^b^	1.00	0.40 (0.21-0.74)	0.50 (0.32-0.79)	0.46 (0.31-0.69)
		Age, gender^c^	1.00	0.52 (0.28-0.97)	0.69 (0.44-1.09)	0.63 (0.42-0.94)
		Age, gender and others^d^	1.00	0.60 (0.27-1.35)	1.21 (0.64-2.26)	0.91 (0.52-1.59)
Both/Both	643	No^b^	1.00	0.59 (0.43-0.79)	0.60 (0.46-0.79)	0.59 (0.48-0.74)
		Age, race and gender^c^	1.00	0.65 (0.48-0.88)	0.76 (0.58-1.00)	0.70 (0.57-0.88)
		Age, race, gender and others^d^	1.00	0.80 (0.57-1.13)	0.97 (0.70-1.34)	0.89 (0.69-1.14)

^a^Except for the final estimates in this column, all RRs and CIs were derived as described in additional file 1: Methods for deriving RR estimates.

^b ^All unadjusted RRs and CIs were derived as described in additional file 1: Methods for deriving RR estimates.

^c^The width of the estimated CI is taken to be the same as the width of the unadjusted estimate, so may be slightly understated.

^d ^The other adjustment factors were year at interview, duration of smoking, cigarettes per day, years since quitting and proportion of years smoked filter cigarettes.

Additional analyses (Table 10) found no increased risk of lung cancer according to the proportion of the known smoking history where mentholated cigarettes were used. Nor was any association seen with long term (> 15 years) use of mentholated cigarettes in the alternative analyses shown in Table 11 that included different subsets of smokers (current smokers, smokers of filter cigarettes, smokers with full information on cigarette type) or involved different assumptions concerning missing data on brand history.

**Table 10:** Risk of lung cancer by proportion of known smoking history smoking mentholated cigarettes among ever smokers - Slone Epidemiology Center case-control study [53]

			Proportion of smoking history		
			
			None	1-49%	50%
			
Gender/Race	No. of cases	Adjusted	RR	RR (CI)	RR (CI)
Both/Both	642	No^a^	1.00	0.86 (0.62-1.18)	0.49 (0.37-0.63)
		Age, race and gender^b^	1.00	0.81 (0.59-1.12)	0.63 (0.48-0.82)
		Age, race, gender and others^c^	1.00	0.86 (0.59-1.24)	0.89 (0.65-1.22)

^a ^All unadjusted RRs and CIs were derived as described in additional file 1: Methods for deriving RR estimates.

^b^The width of the estimated CI is taken to be the same as the width of the unadjusted estimate, so may be slightly understated.

^c ^The other adjustment factors were year at interview, duration of smoking, cigarettes per day, years since quitting and proportion of years smoked filter cigarettes.

**Table 11:** Other estimates for risk of lung cancer for > 15 years of mentholated cigarette smoking - Slone Epidemiology Center case-control study [53]

Analysis	Adjusted	RR (CI)
In current smokers	Age, race, gender and others^a^	0.90 (0.62-1.31)
In smokers of filter cigarettes	Age, race, gender and others^a^	0.95 (0.58-1.58)
In smokers with full information on cigarette type	Age, race, gender and others^a^	0.70 (0.38-1.29)
Assuming smokers smoked mentholated cigarettes where brand history unknown	Age, race, gender and others^a^	0.95^b^
Assuming proportion of menthol use same when brand history not known as when brand history is known	Age, race, gender and others^a^	0.88^b^

^a ^The other adjustment factors were year at interview, duration of smoking, cigarettes per day, years since quitting and proportion of years smoked filter cigarettes.

^b ^CI were not provided.

The authors concluded that: "The results of this study do not support the hypothesis that smoking menthol cigarettes increases the risk of lung cancer relative to smoking nonmenthol cigarettes."

#### Second American Health Foundation multicentre case-control study

The fifth paper providing relevant results [83] was reported in 2003. Unlike the previous papers, which were primarily concerned with risk of lung cancer in relation to mentholated cigarette use, the main objective of this paper was to compare risk in White and Black Americans. As for the first paper [68], it was based on data from the American Health Foundation multicentre hospital case-control study, though the subjects were interviewed between 1984 and 1998 (rather than between 1985 and 1990) and the hospitals involved were not exactly the same. The controls were hospitalized patients with conditions thought not to be associated with smoking, and were matched to the cases on age, gender, hospital and year of interview. The study involved 1964 male and 1484 female histologically confirmed cases interviewed within a year of diagnosis, and 4931 male and 3220 female controls. Information relating to mentholation was limited to whether current smokers preferred mentholated cigarettes or not, with data available for an estimated 963 male and 803 female cases and 1098 male and 572 female controls (assuming this information was available for all the current smokers).

The authors reported RRs (CIs) for menthol preference separately by gender and race, none of which were statistically significant. These were adjusted for age, education, body mass index and pack-years. Table 12 includes these estimates, and also additional derived estimates. The unadjusted estimates, and those adjusted for gender and/or race are all under 1.00. Additional adjustment for age, education, body mass index and pack-years increased the RR somewhat, but none were significant, and most under 1.00. For the overall data, combined over genders and races, the most-adjusted RR estimate was 0.83 (CI 0.68-1.02).

**Table 12:** Risk of lung cancer in current smokers according to preference for mentholated cigarettes - Second American Health Foundation multicentre case-control study [83]

Gender	Race	No. of cases	Adjusted	RR (CI)
Male	White people	799	None^a^	0.79 (0.61-1.01)
			Others^b^	0.83 (0.63-1.09)
	Black people	164	None^a^	0.72 (0.46-1.11)
			Others^b^	1.34 (0.79-2.29)
	Combined	963	None^a^	0.80 (0.65-0.99)
			Race^a^	0.77 (0.62-0.96)
			Race, others^a,b^	0.92 (0.72-1.17)
Female	White people	701	None^a^	0.50 (0.37-0.68)
			Others^b^	0.61 (0.44-1.06)
	Black people	102	None^a^	0.66 (0.38-1.16)
			Others^b^	0.79 (0.41-1.54)
	Combined	803	None^a^	0.52 (0.40-0.67)
			Race^a^	0.53 (0.41-0.70)
			Race, others^a,b^	0.66 (0.46-0.95)
Combined	White people	1500	None^a^	0.66 (0.54-0.80)
			Gender^a^	0.65 (0.54-0.79)
			Others^a,b^	0.76 (0.60-0.96)
	Black people	266	None^a^	0.70 (0.49-0.98)
			Gender^a^	0.70 (0.49-0.98)
			Others^a,b^	1.09 (0.72-1.65)
	Combined	1766	None^a^	0.68 (0.57-0.80)
			Gender, race^a^	0.66 (0.56-0.79)
			Gender, race, others^a,b^	0.83 (0.68-1.02)

^a ^RRs and CIs were derived as described in additional file 1: Methods for deriving RR estimates.

^b ^The other adjustment factors were age, education, body mass index and pack-years.

The authors noted that: "Smokers of menthol flavored cigarettes were at no greater risk for lung cancer than were smokers of unflavored brands."

#### German case-control study

The sixth study with relevant findings was reported as an abstract in 2004 [84]. Unusually, this study was conducted in Germany, in a white population. The study was a hospital-based case-control study involving incident lung cancer cases (839 males and 165 females) and the same number of population controls matched for region, gender and age. Subjects were interviewed about their smoking history including brand names, and ever use of mentholated cigarettes was determined. After adjustment for total amount of tobacco smoking and also for the matching variables, via conditional logistic regression, the RR for ever smoking menthol cigarettes was 1.12 (CI 0.68-1.83). 5% of the cases and 4% of the controls had ever smoked mentholated cigarettes, implying an unadjusted RR of 1.26 (0.83-1.93).

It is not stated in the abstract whether the study was restricted to current smokers or ever smokers or if it included never smokers as well. The analysis would not seem to make sense unless it was restricted to current smokers or ever smokers. As the endpoint is ever use of mentholated cigarettes, it seems likely that the study concerned ever smokers.

The authors comment that "The present study gives no indication for an additional risk of ever smoking mentholated cigarettes if total amount of smoking is taken into account. However, the number of exposed subjects is small hindering definite conclusions with respect to dose."

#### Lung health prospective study

The seventh paper to report results [56] was published in 2007. It was based on data from the Lung Health Study which, in 1986 to 1989, enrolled 3698 male and 2185 female smokers aged 35-60 years with mild to moderate airways obstruction in a clinical trial of smoking cessation. 1961 were randomly assigned to usual care, and 3922 to one of two special interventions (smoking intervention plus either an anticholinergic bronchodilator or a placebo inhaler). The intervention took place over a five-year period, with a follow-up at year 11 and surveillance for mortality to year 14. At baseline and at subsequent annual visits, subjects still smoking were asked "What type of cigarettes are they? Are they plain or menthol?".

The authors reported the results of Cox regression analyses adjusted for age, gender, baseline cigarettes/day, FEV_1 _(as percentage of predicted), randomization group, race and baseline years of education for various causes of death. For lung cancer, based on 240 deaths, the RR for smoking mentholated cigarettes at baseline was estimated as 0.96 (CI 0.70-1.32). No relationship was also seen with mortality from any cause (0.997, 0.83-1.20), coronary heart disease (1.31, 0.77-2.22), or cardiovascular disease (1.03, 0.70-1.52). No further lung cancer RRs can be derived from the data presented.

The authors concluded that "our data contain no evidence that mentholation of cigarettes increases the hazards of smoking."

#### Houston case-control study

The final paper [85], published in 2008, was concerned with the development of a lung cancer prediction model for Black people. It was based on a case-control study conducted in hospitals in Houston from 1995 to 2005. The cases were newly/recently diagnosed, histopathologically confirmed, untreated lung cancers without prior chemotherapy, radiotherapy or recent blood transfusion. Controls, matched to the cases on age, gender and race, came from community centres and a multispecialty physicians' group practice. The analysis focused on cases (294 males and 197 females) and controls (244 males and 253 females) who reported being Black (African-American) people. Analyses of mentholated cigarette use were conducted separately in current smokers and in former smokers.

As shown in Table 13, there was no evidence of an increased risk associated with mentholated cigarette use in any analysis. After adjustment for age, gender and smoking status (current/former smoker), the RR was estimated as 0.81 (0.60-1.09). The authors also noted that, in current smokers, the reduced RR associated with mentholated use (0.69, 0.46-1.03) was non-significant, and remained so after stratification by pack-years. They also reported results of multivariate risk modelling involving a range of risk factors for lung cancer. Variables retained in the final model were smoking status, pack-years of smoking, age at smoking cessation, exposure to asbestos or dusts, and history of COPD or hay fever, but not use of mentholated cigarettes.

**Table 13:** Risk of lung cancer according to mentholated cigarette use - Houston case-control study [85]

Smoking habits	Cases	Adjusted	RR (CI)
Current smokers	278	None^a^	0.63 (0.43-0.93)
		Age, gender	0.69 (0.46-1.03)
Former smokers	176	None^a^	0.76 (0.50-1.17)
		Age, gender	0.99 (0.62-1.56)
Current and former smokers	454	None^a^	0.72 (0.54-0.95)
		Smoking status^a^	0.69 (0.52-0.91)
		Age, gender, smoking status^a^	0.81 (0.60-1.09)

^a ^RRs and CIs were derived as described in additional file 1: Methods for deriving RR estimates.

The authors noted that "In our analysis, we observed no significant risks of lung cancer among former or current smokers who reported smoking mentholated cigarettes (OR range 0.69-0.99), and our data suggested a possible protective effect of mentholated cigarettes for current smokers."

### Study characteristics, strengths and weaknesses

The main features of the eight studies are presented in Table 14 and summarized in Table 15. These features are discussed below, with comments where relevant on the strengths and weaknesses of the studies.

**Table 14:** Main features of the epidemiological studies of cigarette mentholation and lung cancer

Characteristics	1. American Health Foundation study	2. Kaiser Permanente study	3. Los Angeles County study	4. Slone Epidemiology Center study
Source	Kabat and Hebert (1991) [68]	Sidney *et al. *(1995) [51]	Carpenter *et al. *(1999) [69]	Brooks *et al. *(2003) [53]
Study design	Hospital case-control	Prospective cohort	Population case-control	Hospital case-control
Location	USA; New York, Chicago, Detroit, Philadelphia	USA; Oakland, California	USA; Los Angeles County, California	USA; New York, Philadelphia, Massachusetts, Maryland
Timing	1985-1990	1979-1985 followed to 1991	1991-1994	1981-2000
Gender	Both	Both	Both	Both
Age	Unrestricted	30 to 89 years at baseline	40 to 84 years	40 to 74 years
Smoking	Current smokers	Current smokers (for 20+ years)	Ever smoked	Ever smoked (for 20+ years)
Other inclusion criteria	None stated	None stated	Caucasian (non-Hispanic) or African-American people; no previous cancer	Menthol details for 60% of smoking history; no history of cancer
Cases	1044 (588M, 456F)	318 (168M, 150F)	337 (202M, 135F)	643 (435M, 208F)
Cases in mentholated cig smokers	259	93	151	114
Definition of cases	Histologically confirmed, interviewed within 2 months of diagnosis	Incident	Histologically confirmed, interviewed within 7 months of diagnosis	Confirmed by pathology, interviewed within 12 months of diagnosis
At risk	Not applicable	5771M, 5990F	Not applicable	Not applicable
Controls	1324 (914M, 410F)	Not applicable	478 (349M, 129F)	4110 (2123M, 1987F)
Definition of controls	Diseases unrelated to smoking	Not applicable	Licensed drivers (age < 65) and Medicare beneficiaries (age 65+)	Diseases unrelated to smoking
Matching of controls	Age, gender, race, hospital, date of interview	Not applicable	Age, gender, race	No matching
Menthol variable	Time used (< 1, 1-14, 15+ yrs)	Time used (0, 1-9, 10-19, 20+ yrs)	Pack-years menthol (0, 1-15, 16-31, 32+), % menthol (0, 1-19, 20-74, 75-100), cig type (regular only, menthol only, mixed)	Time used (0, 1-15, > 15 years), % years smoked (0, 1-49, 50-100)
Adjustment for race	Yes	Yes	Yes	Yes
Adjustment for smoking habits	Cigs/day, inhalation, duration	Cigs/day, duration	Total pack-years, years since quitting	Cigs/day, duration, years since quitting, time used filter cigarettes
Adjustment for other variables	Age, gender, education, body mass index	Age, gender, education	Age, gender^a^	Age, gender, year of interview
Results by histological type	Squamous cell, small cell, large cell, adenocarcinoma	No	No	No
Study quality^b^	5	9	7	6

Characteristics	5. Second American Health Foundation study	6. German study	7. Lung Health study	8. Houston study

Source	Stellman *et al. *(2003) [83]	Jöckel *et al. *(2004) [84]	Murray *et al. *(2007) [56]	Etzel *et al. *(2008) [85]
Study design	Hospital case-control	Population case-control	Prospective clinical trial	Population case-control
Location	USA; New York, Chicago, Hines, Detroit, Philadelphia	Germany; location not known	USA and Canada; 10 centres	USA; Houston
Timing	1984-1998	Not known	1986-1989 followed for 14 years	1995-2005
Gender	Both	Both	Both	Both
Age	Unrestricted	Unrestricted	35 to 60 years at baseline	Unrestricted
Smoking	Current smokers	Ever smokers^c^	Current smokers	Ever smokers
Other inclusion criteria	None stated	None stated	Mild or moderate airways obstruction, no defined exclusion^d^	Black people
Cases	1766 (963M, 803F)	1004 (839M, 165F)	240M+F	454M+F
Cases in mentholated cig smokers	328	50	About 50	198
Definition of cases	Histologically confirmed, interviewed within 12 months of diagnosis	Incident	Cause of death	Histologically confirmed, newly diagnosed, untreated, with no prior chemotherapy, radiotherapy or recent blood transfusion
At risk	Not applicable	Not applicable	3698M, 2185F	Not applicable
Controls	1670 (1098M, 572F)	1004 (839M, 165F)	Not applicable	353M+F
Definition of controls	Diseases unrelated to smoking	Population	Not applicable	Population
Matching of controls	Age, gender, hospital, year of interview	Age, gender, region	Not applicable	Age, gender, race
Menthol variable	Preference for menthol	Ever smoked mentholated cigs	Smoked mentholated cigs at baseline	Used mentholated cigarettes
Adjustment for race	Yes	Subjects White people	Yes	Subjects Black people
Adjustment for smoking habits	Pack-years	Total amount smoked	Baseline cigs/day	Current/former
Adjustment for other variables	Age, gender, body mass index, education	Age, gender, region	Age, gender, FEV_1_, randomization group, education	Age, gender^e^
Results by histological type	No	No	No	No
Study quality^b^	6	5	8	6

^a^Adjustment for other variables (fruits, vegetables, occupational exposures, family history, ETS) had no appreciable influence, so these variables were not included in the regression models.

^b ^The studies were scored for study quality using NOS scores [81]. Fuller details are available in additional file 2: Study quality.

^c ^Unclear - see text describing this study.

^d^Exclusions were serious illness, pregnant, usual physician prescribed bronchodilators, beta-adrenergic antagonists of systemic glucocorticoids or admitted to drinking in excess of 25 drinks a week.

^e^Multivariate modelling was conducted involving a range of smoking variables, environmental exposures, personal and family history of diseases, but the final model did not include use of mentholated cigarettes.

**Table 15:** Summary of the main features of the epidemiological studies of cigarette mentholation and lung cancer

Characteristic	Level	N (%)	Characteristic	Level	N (%)
Study design			Cases in mentholated cigarette smokers		
	Prospective cohort	2 (25.0)		50-124	4 (50.0)
	Hospital case-control	3 (37.5)		125+	4 (50.0)
	Population case-control	3 (37.5)			
			Controls matched on age and gender		
Country				Yes	5 (62.5)
	USA	7 (87.5)		No	1 (12.5)
	Germany	1 (12.5)		Prospective	2 (25.0)
First year of study			Menthol dose-response studied		
	1979 to 1985	3 (37.5)		Yes	4 (50.0)
	1986 to 1995	4 (50.0)		No	4 (50.0)
	Not known	1 (12.5)			
			Race accounted for		
Year of publication				Yes	8 (100.0)
	1991 to 2000	3 (37.5)			
	2001 to 2008	5 (62.5)	Smoking adjusted for		
				Yes	8 (100.0)
Genders studied					
	Both	8 (100.0)			
			Age adjusted for	Yes	8 (100.0)
Smoking groups used in analysis					
	Current	4 (50.0)	Results by histological type	Yes	1 (12.5)
	Ever	4 (50.0)		No	7 (87.5)
Cases studied					
	200-499	4 (50.0)	NOS study quality score	7 to 9	3 (37.5)
	500-999	1 (12.5)		5 to 6	5 (62.5)
	1000+	3 (37.5)			

#### Study type

The eight studies covered the three most common types of design used in epidemiological research, with two prospective cohort studies [51,56], three hospital case-control studies [53,68,83] and three population case-control studies [69,84,85]. One of the cohort studies [56] was based on follow-up subjects entering a clinical trial. The prospective design virtually excludes the possibility of recall bias. However, both such studies based their analysis on use of mentholation at baseline, which could be over 10 years before the lung cancer occurred, and might have changed during the follow-up period. Neither of these studies concerned representative samples. The clinical trial [56] concerned subjects with mild or moderate airways obstruction, while subjects in the Kaiser Permanente study [51] had to attend for multiphasic health check-up and were noted to be somewhat more educated than the local population and under-representative of the extremes of wealth and poverty. However, any differential risk of mentholated and non-mentholated cigarettes seems unlikely to vary by education, income or prevalence of airway obstruction, so bias should not occur. While case-control studies suffer from the problem that exposure is determined after onset of disease, it is unclear, however, why accuracy of reporting relative use of mentholated and non-mentholated cigarettes should differ materially between lung cancer cases and controls.

#### Location

All but one of the studies were conducted in the United States. The study in Germany [84], reported as an abstract, involved a population where mentholated cigarette use is quite low, and its results, based on a relatively small number of lung cancer cases in mentholated cigarette smokers, contribute relatively little to the meta-analyses described later.

#### Timing

The studies considered started between 1979 and 1995. Inasmuch as mentholated cigarettes only reached an appreciable market share in the 1960s and 1970s, one might expect studies starting later to have more chance of detecting possible effects. However, data by period of mentholated cigarette use were only reported in the first four reported studies [51,53,68,69], limiting the ability to determine lung cancer risk for very long term (e.g. 40+ years) use. However, any major difference in risk between the two types of cigarette might be expected to emerge in the studies so far conducted.

#### Possible overlaps between the studies

It is clear from the information on study location and timing in Table 14, that double-counting of cases is only a possible problem for the three hospital case-control studies. Though the range of years and hospitals varied between the studies, it is likely that there was some overlap in the patients considered. Without reference back to the original data, there is no completely satisfactory solution to this. To avoid loss of power, our main analyses include the data from all the studies, ignoring the overlap, though it is recognized that this may slightly overstate statistical significance. However, as a sensitivity test, some analyses were also run excluding the results from two of these three studies, the first American Health Foundation Study [68] and the Slone Epidemiology Center Study [53], only retaining results from the second American Health Foundation Study [83], the study with the largest number of lung cancer cases in mentholated cigarette smokers.

#### Number of cases and power to detect an effect

The number of cases considered in the analyses ranged from 240 in the Lung Health study [56] to 1766 in the second American Health Foundation multicentre study [83]. Four studies involved over 500 cases and three over 1000. All studies involved both males and females. The proportion of those cases which reported use of mentholated cigarettes was only 4% in the German study [84] but was much higher in the US studies, varying from about 20% in three studies [53,56,83] to over 40% in two [69,85]. This variation would depend on the actual questions asked and where the study was conducted. One of the studies [85] was conducted in Black people. Based on the most adjusted results for the total population studied, all the US studies would have detected as significant, at the 95% confidence interval, an excess risk associated with mentholated cigarette smoking of between 20% and 40%. The German study was less powerful and would only have detected an excess risk of about 65%.

#### Adequacy of the cases

Five of the case-control studies confirmed the diagnosis by histology/pathology, but the German case-control study [84] gave no details. In all these studies, the lung cancer was recently diagnosed. One of the cohort studies [51] gave no information regarding diagnosis of their incident cases, but the other [56], which only considered deaths, classified cause of death based on a review of death certificates, autopsy reports, relevant medical records, and interviews with attending physicians. There is abundant evidence that an in-life diagnosis of lung cancer is unconfirmed by autopsy diagnosis in a moderate proportion (perhaps 10% or so) of cases [86]. Since only one of the studies [56] seemed to consider autopsy evidence, it is likely that some of the cases included were false-positives. However, the procedures taken in most, if not all, of the studies would have greatly reduced this possibility. Although knowledge of smoking habits may affect the likelihood of a lung cancer being detected in life [87,88], it seems implausible that knowledge of mentholation status would do so. It is therefore unlikely that inaccuracy of diagnosis will have had any material effect in these studies.

#### Adequacy of the controls

The three hospital case-control studies [53,68,83] used patients with diseases unrelated to smoking as controls. The descriptions of the controls used were as follows:

**American Health Foundation Study **[68] "Controls were hospitalized patients with conditions thought not be associated with smoking, including: cancers (of the colon, stomach, female breast, prostate, and skin, as well as leukaemia, lymphoma, sarcomas, etc.); benign neoplastic diseases; and non-neoplastic conditions (such as musculoskeletal and connective tissue disorders, eye conditions, injuries, etc.)."

**Slone Epidemiology Center study **[53] "Controls had been admitted for conditions judged to be unrelated to cigarette smoking. The most common control diagnoses included cancers or benign tumors of the breast, colon and rectum, prostate, and other non-tobacco-related sites (45 percent); diseases of the digestive (14 percent), genitourinary (10 percent), or musculoskeletal (7 percent) systems; and injury (13 percent). Respiratory or upper gastrointestinal conditions, regardless of etiology, were excluded."

**Second American Health Education Study **[83] "Eligible control diagnoses excluded tobacco-related diseases such as coronary heart disease, stroke, peripheral vascular disease, chronic obstructive pulmonary disease, gastric ulcer, cirrhosis of the liver, and cancers of the mouth, larynx, esophagus, bladder, kidney, pancreas, or liver." They go on to note the percentage of male and female controls with specific diseases.

When discussing the first of these three studies [68], Sidney *et al. *[51] claimed that "some of these conditions might have been associated with menthol use, obscuring an association with lung cancer". However, it seems rather unlikely that a disease that is not affected by smoking non-mentholated cigarettes would be affected by smoking mentholated cigarettes. Risk of some of the cancers (stomach, breast, and perhaps colon) may, according to recent evidence, be moderately associated with smoking. However, as they form only a proportion of the total controls, and may not be related to menthol use anyway, it seems unlikely that their inclusion would have caused material bias.

Of the three population case-control studies [69,84,85], the one conducted in Germany [84] was reported only as an abstract with no details given of how the controls were derived.

In the Los Angeles county study [69], the population controls used were derived from registers of licensed drivers aged under 65 and of Medicare Beneficiaries aged over 65. However, there seems no guarantee that cases aged under 65 could drive or that cases aged over 65 used Medicare. If ability to drive or use of Medicare is associated with use of mentholated cigarettes, some bias might occur.

In the Houston study of Black people [85], the cases and controls were drawn only from the metropolitan area of Houston. While the cases were recruited "from the University of Texas M.D. Anderson Cancer Center and the Michael E. De Bakey VA Medical Center", the controls were recruited "from Houston area community centers and the Kelsey-Seybold Clinic, Houston's largest multispeciality physicians group practice". It is not apparent how representative the controls were of the population from which the cases were drawn.

In both these studies there was no clear difference in response rate between cases and controls. This was stated to be about 75% in both cases and controls in the Houston study [85] and can be estimated as 70% in cases and 75% in controls in the Los Angeles County study [69]. Response rates were not given for the German study [84] or for two of the case-control studies [53,68], but in the second American Health Foundation study [83], it was noted that "approximately 85% of eligible patients who were approached agreed to be interviewed", though separate figures for cases and controls were not given.

#### Reliability of the data collected

All the studies are limited by possible inaccuracies in the reporting of smoking history and mentholation status. Random errors tend to bias relative risk estimates towards 1.0 and reduce the power of the study to detect a true effect. However, errors may not be random. For example, subjects may tend to think their past habits were more like their current habits than they actually were.

The source of the data on mentholation is summarized in Table 16. As can be seen, this varies from study to study. Four of the studies [51,53,68,84] were asked about brand smoked. In two of these studies [51,53], subjects were also asked whether they smoked mentholated cigarettes but no mention is made of any cross-check being made with the brands reported. One of the studies reporting results for ever smokers [53] only asked about the most recent brand and that used for the longest period, resulting in incomplete information on mentholated cigarette use for many subjects.

**Table 16:** Source of data on mentholation status in the lung cancer studies

Study	Data collected
American Health Foundation study [68]	Subjects were asked about lifetime history of brands of cigarettes smoked (up to 7 brands per person) and information was obtained on whether all brands reported were mentholated or not. If the brand name could not be recalled, the mentholation status was recorded.
Kaiser Permanente study [51]	Subjects were asked about the brand of the cigarette currently smoked and on whether the brand was mentholated or not. The authors did not report that they had obtained information on which brands were mentholated, or that they had checked one answer against the other.
Los Angeles County study [69]	Mentholated cigarette use was based on the question "On average over your lifetime, out of every 100 cigarettes you smoked, how many were menthol?"
Slone Epidemiology Center study [53]	Subjects were asked about the most recent brand and the brand used for the longest period of time to determine the brand name and whether it was mentholated or not. The consistency of the brand name and menthol status was not checked. In some analyses, cigarettes smoked prior to 1956 were assumed not to be mentholated.
Second American Health Foundation study [83]	Details are not given in the methods, but the variable analyzed related to whether current smokers preferred mentholated cigarettes.
German study [84]	Subjects were asked about their smoking history including brand names, with exposure to mentholated cigarettes derived from the brand names blinded for case-control studies.
Lung Health study [56]	At baseline subjects were asked "Do you now smoke cigarettes?" followed by "What type of cigarettes are they? Are they plain or menthol?" Similar questions were asked at annual follow-ups but the answers were not used in the analyses related to risk of lung cancer.
Houston study [85]	Smokers were asked to report their use of mentholated cigarettes, with ever use the endpoint used for analysis.

The effect that error in determining mentholation status might have had on the relative risk estimates is not clear. Inasmuch as mentholated cigarettes have a distinctive taste, any errors may not be too great.

In the Slone Epidemiology Center study [53], subjects with an unknown brand history before 1956 were assumed to have smoked non-mentholated cigarettes at that time. This would probably have led to some over-estimation of the numbers of non-mentholated cigarette smokers and slightly reduced the power to detect effects of mentholation. However, as the assumption applied equally to cases and controls, this is unlikely to have caused material bias.

#### Adjustment for potential confounding variables

Except for the German study [84] and the Houston study of Black people [85], all the studies either adjusted for race, or presented race-specific results from which race-adjusted overall results could be calculated. All the studies adjusted for age, and all either adjusted for gender or presented results from which gender-adjusted overall results could be calculated.

All the studies adjusted for smoking variables, with three studies adjusting for cigarettes per day and duration of smoking [51,53,68] and three studies adjusting for indices of total exposure - pack-years [69,83] or total amount smoked [84]. Some of these six studies adjusted for additional variables: inhalation [68], time used filter cigarettes [53] and in two studies of ever (rather than current) smokers, years since quitting [53,69]. The most limited adjustments are in the Lung Health study [56] which only adjusted for baseline cigarettes/day, the Houston study [85], where results were only available adjusted for current/former smoking, and the German study [84], which appeared to present results for ever smokers but only adjusted for total amount smoked, without taking quitting into account.

Education was adjusted for in four studies [51,56,68,83] and body mass index in two [68,83], with other variables adjusted for being year of interview [53] and FEV_1 _and randomization group [56]. It is of interest that none of the studies adjusted for occupational exposure, which one might expect to vary by mentholated cigarette use (though partially controlled by adjustment for race), and none adjusted for diet.

Generally, the papers did not present results that allowed the reader to determine the effect adjustments for individual variables had on the relative risk estimates. Also, no paper discusses the appropriateness or otherwise of adjusting for smoking characteristics, such as amount smoked or inhalation which may be affected by the choice of brand smoked. There are two conflicting issues here. One is wishing to guard against any potential bias arising if the sort of person who chooses mentholated cigarettes is a more (or less) "addicted" smoker than the sort of person who chooses non-mentholated cigarettes. The other is that if, say, switching to mentholated cigarettes results in an increase in daily consumption with no change in risk per cigarette, adjusting for amount smoked will lead to the impression that mentholation is risk free when it is not. Ideally, the comparison should be between switchers to mentholated cigarettes and non-switchers, adjusted for smoking characteristics *before *the switch, but such analyses seem never to have been attempted.

#### Statistical methods used

Generally, the statistical methods used were standard, with Cox proportion hazards modelling used for the two prospective studies [51,56], and unconditional logistic regression used for all the case-control studies, except the German study [84] which used conditional logistic regression analysis. Four of the case-control studies which used a matched design [68,69,83,85] used unconditional logistic regression, so did not specifically take the matching into account. This was no doubt to avoid loss of power, as some case-control pairs where one subject had never smoked (or in some studies been a former smoker) would not have contributed to a conditional analysis. However, the studies generally took into account most of the matching variables as adjustment variables in analysis. Exceptions were hospital and date of interview in the two American Health Foundation studies [68,83].

#### Results by histological type

Only one study, the first reported [68], gave results by histological type. This is a limitation of the available data.

#### NOS study quality score

Of the two cohort studies, the Kaiser Permanente Study [51] scored 9 out of a possible 9, while the Lung Health Study [56] scored 8, the only weakness being the unrepresentativeness of the cohort, with mild or moderate airways obstruction. All of the six case-control studies scored between 5 and 7, the commonest weaknesses being lack of blind ascertainment of exposure in all six studies, failure to demonstrate that lung cancer cases had been excluded from the controls in four studies, use of hospital controls in three studies, and differing response rates (or lack of information on response rates) in three studies. Wells *et al *[81] do not specify a particular cut-off value for "good quality" studies, and in the meta-analyses following results are compared between those three studies with scores of 7 to 9 and the other five studies with scores of 5 to 6. Fuller details of the study quality scoring are available in additional file 2: Study quality.

### Meta-analysis of results for use of mentholated cigarettes

#### Use of mentholated cigarettes

Table 17 summarizes available RRs and CIs for use of mentholated cigarettes from the eight studies by gender, race and overall. As is evident, the definition of use varies between study, with five studies [53,68,69,84,85] comparing ever and never users of mentholated cigarettes, and three [51,56,83] using alternative comparisons, based on the current or usual brand smoked. The RRs are adjusted for age, gender (where relevant), race (where relevant), smoking habits and other variables. Where the source provides multiple adjusted RRs, those adjusted for the most variables are presented in Table 17. The only individual RRs in the table that are statistically significant at p < 0.05 are the increased risk (RR 1.45, CI 1.03-2.02) in males observed in the Kaiser Permanente study [51], and the decreased risks observed in the Second American Health Foundation study [83] in females (0.66, 0.46-0.95) and in White people (0.76, 0.60-0.96).

**Table 17:** Risk of lung cancer by use of mentholated cigarettes^a ^(adjusted for age, gender, race, smoking habits and other variables^b^)

		RR (CI)				
		
Study	Comparison	Males	Females	White people	Black people	Overall total
American Health Foundation 1 [68]	Ever/never used M (in current smokers)	1.06 (0.82-1.37)	0.78 (0.57-1.08)			0.94 (0.77-1.15)
Kaiser Permanente [51]	Usual brand M or not (in current smokers for 20+ years)	1.45 (1.03-2.02)	0.75 (0.51-1.11)			1.09 (0.85-1.41)
Los Angeles [69]	Ever/never used M (in ever smokers)	1.00 (0.68-1.48)	0.88 (0.50-1.57)	1.02 (0.66-1.58)	0.89 (0.53-1.47)	1.00 (0.72-1.40)
Slone Epidemiology Center study [53]	Ever/never used M (in ever smokers)	0.77 (0.55-1.08)	1.05 (0.72-1.55)	0.93 (0.69-1.24)	0.91 (0.52-1.59)	0.89 (0.69-1.14)
American Health Foundation 2 [83]	Currently prefers/does not prefer M (in current smokers)	0.92 (0.72-1.17)	0.66 (0.46-0.95)	0.76 (0.60-0.96)	1.09 (0.72-1.65)	0.83 (0.68-1.02)
German [84]	Ever/never used M (in ever smokers)			1.12 (0.68-1.83)^c^		1.12 (0.68-1.83)
Lung Health [56]	Current brand M or not (in current smokers)					0.96 (0.70-1.32)
Houston [85]	Ever/never used M (in ever smokers)				0.81 (0.60-1.09)^c^	0.81 (0.60-1.09)
	(in current smokers)				0.69 (0.46-1.03)^c^	0.69 (0.46-1.03)
	(in former smokers)				0.99 (0.62-1.56)^c^	0.99 (0.62-1.56)

^a ^Mentholated cigarettes abbreviated to M in the table.

^b ^All estimates are adjusted for age, gender and race except for those that are gender-specific or race-specific. All estimates are adjusted for smoking habits except for the current and former smoker estimates from the Houston study. See Table 14 for fuller details of adjustment variables.

^c ^Same as the overall results, as the studies were only of White people [84] or Black people [85].

Table 18 gives the results of various meta-analyses. None show significant (p < 0.05) heterogeneity between estimates, and, although random-effects estimates are referred to in the text below, fixed-effect and random-effects estimates are practically the same.

**Table 18:** Meta-analyses of risk of lung cancer by use of mentholated cigarettes

	Number of Estimates	RR (95% CI)			**Heterogeneity χ**^**2**^**(df), p**
			
		Fixed-effect		Random-effects	
**In ever smokers (or current smokers if not available)**					
Overall	8	0.93 (0.84-1.02)		0.93 (0.84-1.02)	4.43 (7), 0.73
Study design					
- prospective	2	1.04 (0.85-1.26)		1.04 (0.85-1.26)	0.38 (1), 0.54
- hospital case-control	3	0.89 (0.78-1.00)		0.89 (0.78-1.00)	0.73 (2), 0.69
- population case-control	3	0.92 (0.76-1.13)		0.92 (0.76-1.13)	1.54 (2), 0.46
NOS study quality score					
- 7 to 9	3	1.03 (0.87-1.22)		1.03 (0.87-1.22)	0.41 (2), 0.84
- 5 to 6	5	0.88 (0.79-0.99)		0.88 (0.79-0.99)	1.94 (4), 0.75
Study size					
- < 125 cases^a^	4	0.99 (0.85-1.15)		0.99 (0.85-1.15)	1.52 (3), 0.68
- 125+ cases	4	0.89 (0.79-1.00)		0.89 (0.79-1.00)	1.59 (3), 0.66
Year of publication					
- 1991 to 2000	3	1.00 (0.86-1.15)		1.00 (0.86-1.15)	0.81 (2), 0.67
- 2001 to 2008	5	0.88 (0.77-0.99)		0.88 (0.77-0.99)	1.82 (4), 0.77
Avoiding possible overlap^b^	6	0.93 (0.83-1.04)		0.93 (0.83-1.04)	4.31 (5), 0.51
Males	5	1.01 (0.88-1.15)		1.01 (0.84-1.22)	7.62 (4), 0.11
Females	5	0.80 (0.67-0.95)		0.80 (0.67-0.95)	3.25 (4), 0.52
White people	4	0.87 (0.75-1.03)		0.87 (0.75-1.03)	2.98 (3), 0.40
Black people	4	0.90 (0.73-1.10)		0.90 (0.73-1.10)	1.30 (3), 0.73
**In current smokers (or ever smokers if not available)**					
Overall	8	0.92 (0.84-1.02)		0.92 (0.84-1.02)	5.70 (7), 0.58
Black people	4	0.88 (0.70-1.10)		0.88 (0.70-1.10)	2.44 (3), 0.49
**In ever smokers specifically**					
Overall	4	0.91 (0.78-1.07)		0.91 (0.78-1.07)	1.60 (3), 0.66
**In current smokers specifically**					
Overall	5	0.91 (0.82-1.02)		0.91 (0.81-1.03)	4.76 (4), 0.31
**In former smokers specifically**					
Total	1	0.99 (0.62-1.56)		0.99 (0.62-1.56)	-

^a ^Lung cancer cases in mentholated cigarette smokers.

^b ^Excluding first American Health Foundation Study and Slone Epidemiology Center study.

**Figure 2:**
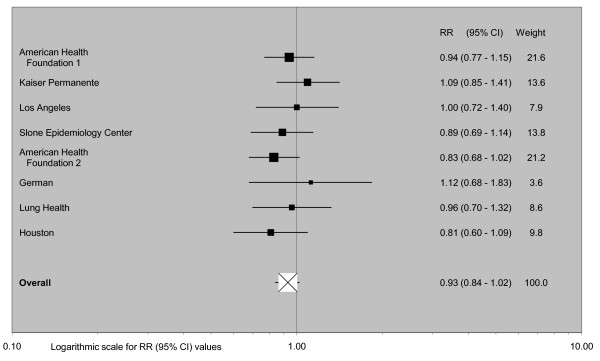
**Forest plot of study-specific estimates and 95% CIs for mentholated cigarette use**. The figure plots the eight combined gender/combined race estimates for use of mentholated cigarettes in ever smokers (or current smokers if not available). Precise definitions of the comparison used for each study are given in Table 17. Each estimate is shown as a square with its area proportional to its weight. The CI is indicated by a horizontal line. The data are plotted on a logarithmic scale so that the estimate is centred in the CI. Also shown in the plot are the actual values of the estimate and its CI and weight. Results from a random effects meta-analysis are also shown. The combined estimate is presented as a diamond with the width corresponding to the CI, and the estimate as the centre of the diamond.

Based on the overall result from each study, and preferring results for ever smokers to results for current smokers where there is a choice (in the Houston study [85]), the meta-analysis estimate (RR 0.93, CI 0.84-1.02) shows no excess lung cancer risk for mentholated over non-mentholated cigarettes (see also Figure 2). This is also true for estimates subdivided by study design, NOS study quality score, study size or year of publication, or when results from the first two hospital case-control studies [53,68] were excluded to avoid possible double-counting of some cases. Five of the studies [51,53,68,69,83] gave gender-specific results with the meta-analysis estimate not significantly increased in males (1.01, 0.84-1.22), despite the significant increase seen in the Kaiser Permanente study [51], and significantly decreased in females (0.80, 0.67-0.95). Three of the studies [53,69,83] gave results separately by race, with one study conducted in White people [84] and one in Black people [85]. The meta-analyses show no evidence of an effect of mentholation in either White (0.87, 0.75-1.03) or Black (0.90, 0.73-1.10) people. Replacing the ever smoker estimate from the Houston study [85] by that for current smokers made little difference to the overall estimate (which became 0.92 (0.84-1.02)), or that for Black people (which became 0.88 (0.70-1.10)). Nor is there any evidence of an effect overall in ever smokers specifically (0.91, 0.78-1.07) or in current smokers specifically (0.91, 0.81-1.03). Only one study provided an estimate for former smokers and that (0.99, 0.62-1.56) also showed no effect.

Only one study [68] gave estimates by lung cancer type, but, as shown in Table 4, there was no evidence of an effect of mentholation for any of the four lung cancer types studied.

Only one study [51] gave estimates by age group. As shown in Table 5, there was no indication of heterogeneity by age, the (gender-specific) RRs for the four age groups being consistent with those for the combined age groups.

Only one study [83] provided estimates jointly by race and gender, but, as shown in Table 12, none of these were statistically significant.

Three of the studies [53,69,83] presented comparable RR estimates that were similarly adjusted for non-smoking variables but were either adjusted or not adjusted for smoking. In the Los Angeles County study [69], adjustment for smoking habits increased the overall RR from 0.87 (0.66-1.15) to 1.00 (0.72-1.40), in the Slone Epidemiology Center study [53], adjustment increased it from 0.70 (0.57-0.88) to 0.89 (0.69-1.14), and in the Second American Health Foundation study [83], adjustment increased from 0.66 (0.56-0.79) to 0.83 (0.68-1.02). As discussed earlier, the extent to which adjustment for smoking habits should be carried out is open to question. However, these results do not suggest that the failure to find an increased risk from mentholated cigarettes depends crucially on smoking adjustment.

**Figure 3:**
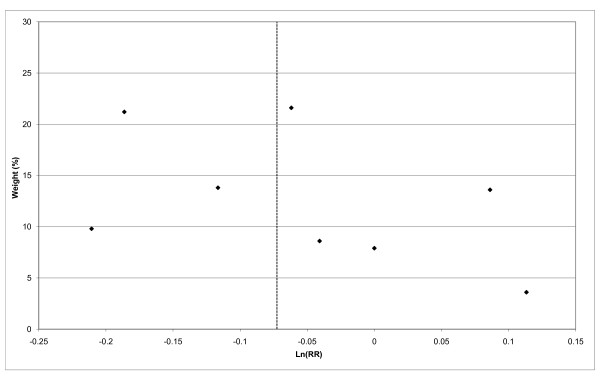
**Funnel plot for risk of lung cancer by use of mentholated cigarettes**. Funnel plot of the eight relative risk estimates for use of mentholated cigarettes and lung cancer shown in Figure 2 against their weight (inverse-variance of log RR.) The dotted vertical line indicates the fixed-effect meta-analysis estimate.

For the principal meta-analysis, for which the data are shown in Figure 2, there was no indication of publication bias, as assessed by Egger's test[82] or by a funnel plot (Figure 3).

### Meta-analysis of results for long-term use of mentholated cigarettes

Table 19 summarizes available RRs and CIs for long term use of mentholated cigarettes from the four studies providing relevant data. The definitions of long term use vary by study, two studies giving results for 15+ years use, one for 20+ years use, and one for 32+ pack-years of use. As for Table 17, the RRs are always the most-adjusted available. None of the individual RRs shown in Table 19 are statistically significant.

**Table 19:** Risk of lung cancer by long-term use of mentholated cigarettes^a ^(adjusted for age, gender, race, smoking habits and other variables^b^)

		RR (CI)				
		
Study	**Exposure**^**c**^	Males	Females	White people	Black people	Total
American Health Foundation 1 [68]	15+ years use of M (in current smokers)	0.98 (0.70-1.38)	0.76 (0.53-1.16)			0.88 (0.68-1.14)
Kaiser Permanente [51]	20+ years use of M (in current smokers for 20+ years)	1.59 (0.96-2.63)	0.70 (0.40-1.23)			1.10 (0.76-1.60)
Los Angeles [69]	32+ pack-years M (in ever smokers)	1.48 (0.71-3.05)	0.41 (0.15-1.11)	1.06 (0.47-2.36)	0.90 (0.38-2.12)	0.95 (0.53-1.70)
						
Slone Epidemiology Center study [53]	15+ years of M (in ever smokers)	0.91 (0.57-1.46)	1.00 (0.63-1.60)	1.01 (0.68-1.51)	1.21 (0.64-2.26)	0.97 (0.70-1.34)

^a ^Mentholated cigarettes abbreviated to M in the table; reference group is those with no use of mentholated cigarettes.

^b ^Details of all adjustment variables are given in Table 14.

**Figure 4:**
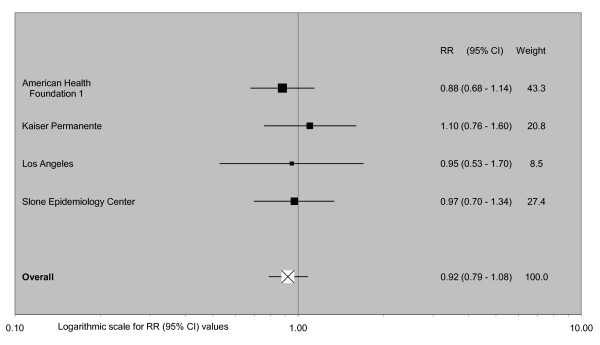
**Forest plot of study-specific estimates and 95% CIs for long-term use of mentholated cigarettes**. The figure plots the four combined gender/combined race estimates for long-term use of mentholated cigarettes in ever smokers (or current smokers if not available). Precise definitions of the comparison used for each study are given in Table 19. Each estimate is shown as a square with its area proportional to its weight. The CI is indicated by a horizontal line. The data are plotted on a logarithmic scale so that the estimate is centred in the CI. Also shown in the plot are the actual values of the estimate and its CI and weight. Results from a random effects meta-analysis are also shown. The combined estimate is presented as a diamond with the width corresponding to the CI, and the estimate as the centre of the diamond.

Table 20 gives the results of meta-analysis overall and by gender and race. None of the random-effects estimates suggest any effect of long term mentholation use on risk of lung cancer, either overall (RR 0.95, CI 0.80-1.13 - see also Figure 4 - or for males (1.13, 0.86-1.47), females (0.78, 0.60-1.01), White (1.02, 0.71-1.46) or Black (0.96, 0.71-1.30) people. However, there are few estimates, four for the overall results and those by gender, and only two for the results by race. No significant heterogeneity was evident in any of the analyses.

**Table 20:** Meta-analyses of risk of lung cancer by long term use of mentholated cigarettes

	Number of Estimates	RR (95% CI)			Heterogeneity ** χ**^**2**^**(df), p**
			
		Fixed-effect		Random-effects	
Overall	4	0.95 (0.80-1.13)		0.95 (0.80-1.13)	0.95 (3), 0.81
Males	4	1.11 (0.88-1.39)		1.13 (0.86-1.47)	3.76 (3), 0.29
Females	4	0.78 (0.60-1.01)		0.78 (0.60-1.01)	2.84 (3), 0.42
White people	2	1.02 (0.71-1.46)		1.02 (0.71-1.46)	0.01 (1), 0.92
Black people	2	1.09 (0.66-1.81)		1.09 (0.66-1.81)	0.30 (1), 0.59

Some of these studies also report other results by extent of use of mentholated cigarettes. In the Los Angeles County Study [69], no excess risk was seen comparing ever smokers with 75-100% mentholated cigarette use with those with 0% use (1.02, 0.65-1.63) or comparing those with exclusive mentholated cigarette use with exclusive regular cigarette use (1.04, 0.62-1.75). Nor were significant effects of mentholation seen when the 32+ pack-years category was subdivided into 32-53 and 54+ pack years, RRs based on limited data being 0.76 (0.37-1.59) and 1.38 (0.56-3.40). In the Slone Epidemiology Center study [53], ever smokers with 50% or more mentholated cigarette use had no excess risk compared to those with no use (0.89, 0.65-1.22). That study also presented estimates of risk for > 15 years use of mentholated cigarettes alternative to that of 0.89 (0.69-1.14) used in Tables 19 and 20. As shown in Table 11, no association was seen with lung cancer risk whether different subsets of smokers were used or whether different assumptions were made concerning data on brand history.

### Can mentholation explain the higher lung cancer risk in US Black people?

It is often suggested, e.g. [17,67-69], that the greater preference of Black people for mentholated cigarettes might help to explain the higher lung cancer rates seen in Black males compared to White males. However, the epidemiological findings would appear to argue otherwise. For mentholation to be the major cause one would expect to see a substantially higher risk of lung cancer in mentholated cigarette smokers, but this is not the case. Table 21 presents approximate calculations suggesting that explaining the 36% increase in incidence and 31% increase in mortality reported in Black males compared to White males [66] requires the relative risk of lung cancer associated with mentholation to be about 1.7 or 1.8. In fact, as shown in Table 18, the relative risk for mentholation estimated from the combined available evidence is 0.93, with an upper 95% confidence limit of only 1.02, far less than 1.7 or 1.8. Even for long term use (see Table 20) the estimate of 0.95 has an upper 95% confidence limit of only 1.13. Though a small contribution of mentholation to the excess risk in US Black people cannot be ruled out, a major contribution seems totally implausible based on the available data. The fact that Black females, compared to White females, are much more likely to use mentholated cigarettes but have quite similar lung cancer mortality, strengthens this view.

**Table 21:** Approximate estimation of the relationship in males between the mentholated/non-mentholated lung cancer relative risk (RR) and the estimated lung cancer risk of Black people relative to White people

		Never smoked	Ex smoker			Current smoker			Total
		Total	Total	Menthol	Non-menthol	Total	Menthol	Non-menthol	Total
		(1)		(2)	(3)		(4)	(5)	
	White people								
	Frequency^1^	0.501	0.262			0.237			1.000
	Menthol proportion^b^			0.218	0.782		0.218	0.782	
(a)	Frequency by menthol	0.501		0.057	0.205		0.052	0.185	1.000
(b)	Risk relative to never smokers^c^	1.00	9.36	9.36RR	9.36	22.36	22.36RR	22.36	
	Black people								
	Frequency^1^	0.572	0.158			0.270			1.000
	Menthol proportion^b^			0.835	0.165		0.835	0.165	
(a)	Frequency by menthol	0.572		0.132	0.026		0.225	0.045	1.000
(b)	Risk relative to never smokers^c^	1.00	9.36	9.36RR	9.36	22.36	22.36RR	22.36	
	Assumed value of RR				Black/White relative risk^d^				
	1.0				0.980				
	1.1				1.035				
	1.2				1.088				
	1.3				1.138				
	1.5				1.234				
	1.8				1.365				
	2.0				1.445				

^a ^See Table 3.

^b ^See Table 1 - proportions assumed the same for current and ex smokers.

^c ^The source of the relative risk estimates of 9.36 for ex smokers and 22.36 for current smokers is the 1989 US Surgeon-General's Report Table 6 p150 [65]. As they are based on Cancer Prevention Study II starting in 1982, a study in a predominantly White population, the relative risk estimates have been assumed to apply to non-mentholated cigarette smokers. RR is the assumed relative risk for mentholated compared to non-mentholated cigarette smoking.

^d ^Estimated by summing for each race, the product of rows (a) and (b) over columns (1), (2), (3), (4) and (5), and then dividing the total for Black people by the total for White people.

## Discussion

As noted earlier, there has been increasing regulatory interest in the possible contribution of additives to the carcinogenicity of cigarettes, both in the United States and Europe. In principle, menthol is by far the easiest to study, as brand names clearly identify whether or not the cigarette is mentholated, and smokers will be well aware anyway whether the cigarettes they are smoking are mentholated. Also mentholated cigarettes are quite widely smoked, particularly in the United States, especially in Black people who have a much greater preference for them than do White people (see Table 1).

While menthol itself has been widely used for many years and experimental studies provide no reason for concern that it is genotoxic or carcinogenic [7,8], there is a suggestion that its acute effects on the mouth, nose and respiratory system [6] may affect how smoke from cigarettes is inhaled [10]. Coupled with evidence in the United States that Black males have markedly higher lung cancer rates than do White males (see Table 2), despite Black people smoking less heavily and tending to start smoking later in life than do White people (see e.g. Table 3), it is often suggested (e.g. [17,67-69]) that mentholation of cigarettes may increase the risk of lung cancer.

The main objective of this paper is to investigate this possibility by a direct epidemiological comparison of risk in mentholated and non-mentholated cigarette smokers. However, it should be noted that various other pieces of evidence argue against this possibility. First, data from a number of studies (see e.g. [9,45]) provide no convincing evidence that mentholation increases puffing, inhalation or tobacco smoke uptake. Also, Black females have similar lung cancer rates (see Table 2) to White females, despite the preference for mentholated cigarettes in Black people being at least as great in females as in males (see Table 1). It should also be noted that, as described in the Background section, there are other differences in smoking characteristics between Black and White people, with Black people being more likely to be current smokers, less likely to quit, tending to choose higher tar cigarettes and having higher cotinine levels. Other differences between Black and White people may also be relevant, for example in body mass index, access to health care and metabolism.

Although mentholated cigarettes have assumed an important place in the United States cigarette market over the last 50 years, and the number of published epidemiological studies of smoking and lung cancer is extremely large, relatively few publications provide information comparing risk in smokers according to use of mentholated cigarettes.

While only eight relevant studies were identified, and two of the papers reporting on this relationship [83,85] did not have mentholation as a central interest and one [84] was published only as an abstract, the data available to study effects of mentholation seem quite good. The studies are reasonably large, involving in total some 1200 lung cancers in mentholated cigarette smokers and are of standard designs analysed by standard methods. Cases are generally histopathologically confirmed, with selection of controls unlikely to cause relevant bias. All the studies take age, gender, race and other aspects of smoking into account in their analyses, with some adjustment for other potential confounding variables such as education and body mass index.

Nevertheless, the studies have limitations. These include failure to present results by histological type (except in one study [68]), failure to adjust for occupation or diet, and failure to report results by length of use of mentholated cigarettes in the later published studies [56,83-85] - important as the earlier studies [51,53,68,69] had insufficient subjects who smoked mentholated cigarettes for a long time. One would like to be able to compare subjects who smoked only mentholated or only non-mentholated cigarettes for 30 years or more.

Another issue is the extent and reliability of the data on lifetime use of mentholated cigarettes. Some studies only collected or analyzed information on brand currently smoked [51,56,83] or on a few brands smoked during lifetime [53], and some studies collecting data on names of brands smoked and whether the brand was mentholated [51,53] seemed not to cross-check this information. The reliability of statements on brands smoked years ago is in any case questionable [89].

The analyses presented are typically adjusted for smoking habits, such as daily cigarette consumption and duration of smoking. This is an attempt to compare mentholated and non-mentholated cigarette smokers with an equivalent smoking history. None of the analyses have attempted to account for the possibility that switching from non-mentholated to mentholated cigarettes might be associated with changes in cigarette consumption.

The combined data from the eight studies are not at all suggestive of any effect of mentholation on lung cancer risk. Meta-analysis of adjusted RRs for ever use give a combined estimate of 0.93 (95% CI 0.84-1.02), with individual estimates showing remarkably little heterogeneity, varying only from 0.81 to 1.12. The same is true for long-term use, where the combined estimate of 0.95 (0.80-1.13) is again based on consistent individual estimates, varying from 0.88 to 1.10. There is also no evidence of an increase in males or females separately, in Black or White people separately or in estimates for ever smokers, current smokers or former smokers. Limited data on risk by age and by histological type of lung cancer also suggest no effect of mentholation. There is a question as to the validity of adjustment for aspects of smoking habits that might be affected by use of mentholated cigarettes, but the estimates that are adjusted only for non-smoking variables tend to be lower. Overall the data, taken as a whole, could hardly be more indicative of a lack of relationship.

For mentholation to explain the increased lung cancer risk in Black compared to White males of some 30-35% would require a relative risk of about 1.7 to 1.8. The excess risk, therefore, cannot possibly be explained by the much greater preference of Black people for mentholated cigarettes.

## Conclusion

While there are some weaknesses in the studies presenting data, discussed in detail in the report, the evidence taken as a whole is certainly consistent with the addition of menthol to tobacco having no effect on the lung carcinogenicity of cigarettes. The much greater preference for mentholated cigarettes in Black people in the United States cannot possibly explain their higher lung cancer risk, which in any case in evident only in men.

## Abbreviations

CI: confidence interval; CO: carbon monoxide; NOS: Newcastle-Ottawa quality assessment scale; RR: relative risk; SCENIHR: Scientific Committee on Emerging and Newly-Identified Health Risks; TPSAC: Tobacco Products Scientific Advisory Committee (TPSAC)

## Competing interests

The author, founder of P.N. Lee Statistics and Computing Ltd., is an independent consultant in statistics and an adviser in the fields of epidemiology and toxicology to a number of tobacco, pharmaceutical and chemical companies.

## Authors' contributions

The author planned the study, and carried out all phases of it, except as indicated in the acknowledgements section.

## Pre-publication history

The pre-publication history for this paper can be accessed here:

http://www.biomedcentral.com/1471-2466/11/18/prepub

## Supplementary Material

Additional file 1 **Methods for deriving RR estimates**. DOC file giving details of the statistical methods used in each study to derive RR estimates from data presented in the source publications. Click here for file

Additional file 2 **Study quality**. XLS file giving full details of the scoring of study quality based on NOS. The file shows the nine component criteria which make up the NOS, the indicator of study quality for each, and the individual component scores used for each study. It also shows where there was a disagreement between the two assessors, and whose assessment was taken, and why. Click here for file
