# Supplementary material for: Systematic review of the epidemiological evidence comparing lung cancer risk in smokers of mentholated and unmentholated cigarettes
Source: BMC Pulm Med. 2011 Apr 18;11:18. doi: 10.1186/1471-2466-11-18 (PMC3103484; doi:10.1186/1471-2466-11-18)
Supplement: Additional file 1 — Methods for deriving RR estimates. DOC file giving details of the statistical methods used in each study to derive RR estimates from data presented in the source publications. [file 1471-2466-11-18-S1.DOC]

**Systematic review of the epidemiological evidence comparing lung cancer risk in smokers of mentholated and unmentholated cigarettes**

**APPENDIX A**

Details of methods used to estimate RRs and CIs

**Abbreviations used**

CI Confidence interval

FM Fixed-effect meta-analysis

HAM Method of Hamling *et al.*

RR Relative risk

STM Standard methods

The RRs and CIs relating mentholation use to lung cancer used in the paper are derived using various methods as described below.

**American Health Foundation multicentre case-control study [68]**

Table 4

The source paper (Tables 3 and 4) gives all the adjusted (RR (CI) values for 1-14 and 15+ years use, except for the combined gender results for any histological type which were derived by fixed-effect meta-analysis (FM) from the gender-specific estimates.

Adjusted RR (CI) values for ever use were derived from the values by years use using the method of Hamling *et al.* (2007) [80] (HAM).

All unadjusted RRs and CIs are derived from the total number of cases and controls and distribution (%) of menthol use given in Table 1 of the source using standard methods (STM) described in e.g. Chapter 6 of Gardner and Altman (1989) [78]. Again, FM was used for combined gender results.

**Kaiser Permanente prospective study [51]**

Table 5

All the gender-specific RRs and CIs are given in the source paper (Tables 2, 4 and 5) except that the estimates adjusted only for age were derived by FM from the age-specific estimates.

All the gender-combined estimates were derived by FM from the gender-specific estimates.

**Los Angeles county case-control study [69]**

Table 6

The source (Tables 2, 3 and 4) give all the adjusted RRs for 1-15, 16-31 and 32+ pack-years, with CIs also for the most-adjusted. It also gives numbers of cases and controls from which unadjusted RRs and CIs can be derived by STM. CIs for the lesser-adjusted RRs have been calculated approximately assuming that the widths of the CIs are the same as for the unadjusted RRs.

RRs and CIs for ever use were derived from the pack-years values using STM for the unadjusted estimates and HAM for the adjusted estimates.

Tables 7 and 8

These were derived from the source data in Table 2 using the same methods as for Table 6.

**Slone Epidemiology Center study [53]**

Table 9

The source (Tables 2 and 3) give all the adjusted RRs for 1-15 and >15 years smoked, with CIs also for the most-adjusted. It also gives numbers of cases and controls from which unadjusted RRs and CIs can be derived by STM. CIs for the lesser-adjusted RRs have been derived assuming that the widths of the CIs are the same as for the unadjusted RRs.

The same applies to the ever use RRs and CIs given in Table 2 for the combined gender/race analysis. The gender- and race-specific RRs and CIs for ever use were derived from the years smoked values using STM for the unadjusted estimates and HAM for the adjusted estimates.

Table 10

The source is Table 2, and the same methods were used to derive RRs and CIs as were used in Table 9 for the combined gender/race analysis.

Table 11

These estimates are all taken from the text of the source paper.

**Second American Health Foundation multicentre case-control study [83]**

Table 12

The source (Table 4) gives fully adjusted RR (CI) values by gender and race. These were combined over race, gender or both by FM.

All unadjusted RRs and CIs were calculated using STM from the total number of cases and controls and the distribution (%) by smoking habits and by menthol preference within current smokers given in Table 2 of the source. Again, FM was then used to derive estimates adjusted for race, gender or both.

**German case-control study [84]**

The unadjusted RR and CI was estimated from the total number of cases and controls and the percentage ever use of mentholated cigarettes given in the abstract using STM.

**Lung health prospective study [56]**

No estimates were derived; the only estimate used being that given in Table 4 of the source.

**Houston case-control study [85]**

The source (Table 2) gives age- and gender-adjusted RR (CI) values by smoking status (current or former). These were combined over smoking status by FM.

Unadjusted RRs and CIs were derived from the numbers and percentages of mentholated cigarette smokers by smoking status given in Table 1 using STM. The unadjusted RRs were combined over smoking status by FM.
